# Compatibility Review for Object Detection Enhancement through Super-Resolution

**DOI:** 10.3390/s24113335

**Published:** 2024-05-23

**Authors:** Daehee Kim, Sungmin Lee, Junghyeon Seo, Song Noh, Jaekoo Lee

**Affiliations:** 1 NAVER Cloud Corp., Seongnam 13529, Republic of Korea; 2 College of Computer Science, Kookmin University, Seoul 02707, Republic of Korea; 3 SK Telecom, Seoul 04539, Republic of Korea; 4 Department of Information and Telecommunication Engineering, Incheon National University, Incheon 22012, Republic of Korea

**Keywords:** deep learning, neural networks, super-resolution, object detection, face recognition

## Abstract

With the introduction of deep learning, a significant amount of research has been conducted in the field of computer vision in the past decade. In particular, research on object detection (OD) continues to progress rapidly. However, despite these advances, some limitations need to be overcome to enable real-world applications of deep learning-based OD models. One such limitation is inaccurate OD when image quality is poor or a target object is small. The performance degradation phenomenon for small objects is similar to the fundamental limitations of an OD model, such as the constraint of the receptive field, which is a difficult problem to solve using only an OD model. Therefore, OD performance can be hindered by low image quality or small target objects. To address this issue, this study investigates the compatibility of super-resolution (SR) and OD techniques to improve detection, particularly for small objects. We analyze the combination of SR and OD models, classifying them based on architectural characteristics. The experimental results show a substantial improvement when integrating OD detectors with SR models. Overall, it was demonstrated that, when the evaluation metrics (PSNR, SSIM) of the SR models are high, the performance in OD is correspondingly high as well. Especially, evaluations on the MS COCO dataset reveal that the enhancement rate for small objects is 9.4% higher compared to all objects. This work provides an analysis of SR and OD model compatibility, demonstrating the potential benefits of their synergistic combination. The experimental code can be found on our GitHub repository.

## 1. Introduction

Deep learning (DL) has facilitated tremendous progress in the computer vision domain in recent years. Cutting-edge results have been consistently achieved using public benchmark datasets such as ImageNet [1], Pascal VOC [2], and MS COCO [3]. As new challenges emerge in Kaggle competitions, researchers are increasingly focused on applying successful DL models to real-world applications, including autonomous driving, visual inspection, robotics, medical image analysis, and masked face recognition [4,5].

Object detection (OD) is a critical technique for addressing various computer vision problems. The development of detection networks, such as Faster R-CNN [6] and You Only Look Once (YOLO) [7], has enabled the practical implementation of vision applications with exceptional performance and rapid processing capabilities. Nonetheless, a fundamental limitation exists that cannot be resolved solely by OD: performance degradation when image quality is low, e.g., in the presence of image noise [8]. This performance degradation is particularly pronounced when detecting small objects [9]. We found that employing image super-resolution (SR) to convert low-quality input images into high resolution can overcome this issue, analogously to how wearing glasses can improve poor vision [9].

Although previous studies [10,11] have applied SR to OD tasks, an in-depth analysis of the fusion of various SR architectures and object detectors remains unexplored. To address this, we investigate well-known DL-based SR models and representative OD models reported in the literature, assessing the compatibility of these models for detailed profiling. The selection criteria for SR models involved classifying neural network models based on their architectural characteristics and selecting high-performance models from each group, using peak signal-to-noise ratio (PSNR) and structural similarity index measure (SSIM) [12] as performance indicators. We conducted comprehensive experiments with the MS COCO [3] and Widerface [13] datasets, examining the performance change in object detectors according to the SR method and quantifying the improvement in object detector performance using the SR method.

In this paper, we start with the definition of the single-image SR task (Section 3), followed by the classification of major SR models (Section 4) and OD models (Section 5) based on their structural characteristics, providing a brief explanation of their architectural components. We then compare the OD performance improvement of well-known SR and OD models (Section 6), summarizing and sharing key findings from the experimental results (Section 7). In our experiments, we employ multiple degradation methods to simulate various low-resolution image conditions by applying various degradation methods to the original images. As a result, our experiment showed that the OD performance increased when the SR method was applied to the low-resolution image, and it was confirmed that, in particular, the performance improvement was the largest for small objects. The main contributions of this study are as follows:

- We observe that, as the PSNR and SSIM values of the SR models increased, the performance improvement rate of the OD models also increased. Interestingly, even when the PSNR and SSIM values were reduced for SR models utilizing adversarial learning, OD performance still increased.
- We introduce the performance enhancement rate as a metric to empirically analyze the compatibility between SR models and object detectors based on their structural features. Our analysis reveals that pre-upsampling-based SR models have negligible impact, and transformer-based object detectors exhibit higher compatibility with SR models than other detection models. This insight is expected to guide future research in addressing the limitations of transformer-based detectors.
- We conducted experiments using the latest OD model and SR models like the transformer-based ones. In the recent transformer-based SR models, despite improvements in PSNR and SSIM metrics for bicubic interpolation (BI) degradation, a decrease in OD performance was observed. Conversely, OD performance improvements were noted for both blur downscale (BD) and downscale noise (DN) degradation. For the recent transformer-based object detector, such as DETR, performance augmentation was indeed observed. However, the extent of performance improvement, when compared to alternative models, remained modest.
- We provide experimental evidence according to which, even when SR models are trained independently, combining them with OD models can improve OD performance.

## 2. Background

SR, a low-level computer vision task, aims to enhance image or video quality. In recent years, research has explored the interconnectedness of SR and high-level computer vision tasks. For example, Pang et al. [14] demonstrated improved small-scale pedestrian detection by jointly training the SR and classification modules. Wang et al. [15] proposed a method to enhance semantic segmentation performance by training the SR and semantic segmentation modules concurrently. Xiao et al. [16] proposed a method of applying video super-resolution (VSR) to online video. Ju et al. [17] proposed super-resolution photometric stereo network (SR-PSN) to acquire high-resolution 3D surface structures.

There are two main branches for combining OD and SR: directly applying SR on the image, and applying SR on features extracted from the detector backbone. Various methods have been proposed for SR on input images [11,18], while Zheng et al. [19] used deblurring to improve the input image. Moreover, SOD-MTGAN [10] utilized SR for regions of interest (RoIs). On the other hand, Noh et al. [20] applied SR to features extracted from the backbone.

In the literature, most studies trained models jointly. However, if performance improvement occurs without joint training, it has the advantage of being easy to use by attaching two models. This is also due to the fact that the training recipe becomes rather complicated if models are trained jointly. Thus, in this study, we will experiment with vanilla combinations (i.e., SR–OD) that confirm the performance improvement only from the structural point of view of the SR and OD models. With a good combination of the performance improvement confirmed here, we expect to be able to achieve greater performance improvement through joint training later.

## 3. Single Image Super-Resolution Methods

An SR method can be approached differently depending on whether the input data type is a video or a single image. The scope of SR methods in this study is limited to single-image SR (SISR) to determine whether SR improves the detection performance of an object detector.

Conventional SR methods can primarily be classified as example-based, reconstruction-based, and interpolation-based [21]. Example-based SR methods, which show the best performance among the previously mentioned methods, are also referred to as learning-based methods because they are based on machine learning. To capture the relationship between LR and HR images, example-based SR methods learn a mapping function based on machine learning methods such as sparse representation [21], local linear regression [22], and random forest [23].

Example-based SR methods have evolved to train DL-based SISR models based on a rich HR–LR image pair dataset.

(1) $$\begin{array}{c} I_{LR}=D\left(I_{HR}+n_{1}\right)+n_{2} \end{array}$$

where *D* and *n* denote a downsampling method and noise, for Gaussian noise, blur ⊂n. An LR image $I_{LR}$ of a dataset is commonly generated via a downsampling method, such as bicubic interpolation in the original HR image $I_{HR}$, as shown in Equation (1). Well-known degradation methods commonly used in SR experiments include BI, BD, and DN. BI generates LR output through downsampling only with bicubic interpolation. BD blurs an HR image using a 7 × 7 Gaussian kernel with a standard deviation of 1.6 and generates an LR image through BI. DN generates a temporary LR image through BI degradation via the addition of Gaussian noise at noise level 30 to the generated LR image [24]. Complex degradation that involves BD or DN is employed to simulate a more complex image as a real image is more complex than that obtained by simply using BI.

The DL-based SR model is highly dependent on the performance of the degradation method as it learns a mapping function that reverses the degradation method (i.e., from LR to HR). Accordingly, recent studies have been proposed to generate LR images similar to reality by compounding degradation such as BI, BD, and DN [25,26].

(2) $$\begin{array}{c} I_{SR}=N\left(I_{LR}\right) \end{array}$$

The general principle of a CNN-based SR model is shown in Equation (2). An SR model *N* generates an image $I_{SR}$ of the same resolution as that of the original image $I_{HR}$ using downsampled $I_{LR}$ as its input and learns to equalize it to $I_{HR}$. *N* learns LR–HR denoising and mapping functions through this process and can generate an HR image robustly, even when a new LR image is used as input.

Initial models, such as the SR convolutional neural network (SRCNN) [27], have been used to simulate the conventional SR algorithm using a simple CNN model. With the advancement of CNN models, studies for SR tasks, such as VDSR [28], based on deeper and more complex models [29], have been proposed. Recently, various DL architectures have been applied to SR tasks, such as the super-resolution generative adversarial network (SRGAN) [30]. At the same time, studies to implement the CNN architecture for sparse coding and the reference-based approach, which are traditional concepts employed before DL, such as SCN [31] and CrossNet [32], have been conducted.

### Upsampling Methods in SISR

Upsampling methods have the most significant impact on SR model performance as they restore LR images to HR images. The effect of these methods varies depending on the timing of the application and how the upsampling method is implemented [9].

The structure according to the upsampling position of an SR model is shown in Equations (3) and (4). Here, *U* denotes the upsampling function, *C* denotes the convolution filter, including the bias term, activation function, and batch normalization (BN), and *i* denotes the index of the layer.

(3) $$\begin{array}{c} N_{pre}=U\left(I_{LR}\right)*C_{1}*C_{2}*\ldots *C_{i} \end{array}$$

(4) $$\begin{array}{c} N_{post}=U\left(I_{LR}*C_{1}*C_{2}*\ldots \right)*C_{i} \end{array}$$

Initial DL-based SR models, including SRCNN [27] or VDSR [28], were primarily pre-upsampling models that used interpolation to enlarge input images, as shown in Equation (3). However, one disadvantage of pre-upsampling models is that their operation is inefficient, which is why post-upsampling models, such as FSRCNN [33] and ESPCN [34], upsample the feature map at the end of the network, as shown in Equation (4). However, it was pointed out that these post-upsampling models are not advantageous for obtaining good results as they perform upsampling only once.

(5) $$\begin{array}{c} N_{prog}=U_{n}\left(\ldots \left(U_{1}\left(I_{LR}*C_{1}*\ldots \right)*C_{m}*\ldots \right)\right)*C_{i} \end{array}$$

(6) $$\begin{array}{c} N_{iter}=U_{n}\left(D_{n-1}\ldots \left(D_{1}\left(U_{1}\left(I_{LR}*C_{1}*\ldots \right)\right)\right)\right)*C_{i} \end{array}$$

A progressive upsampling (reconstruction) method, such as $N_{prog}$ of Equation (5), which performs upsampling through several stages, as in the Laplacian pyramid SR network (LapSRN) [35], is proposed to solve this problem. Furthermore, an iterative upsampling and downsampling model, which performs upsampling and downsampling (*D*) recursively, rather than upsampling only once, is also proposed, as shown in $N_{iter}$ of Equation (6). A representative example is DBPN [36].

## 4. Taxonomy of Super-Resolution Architectures

**Figure 1:**
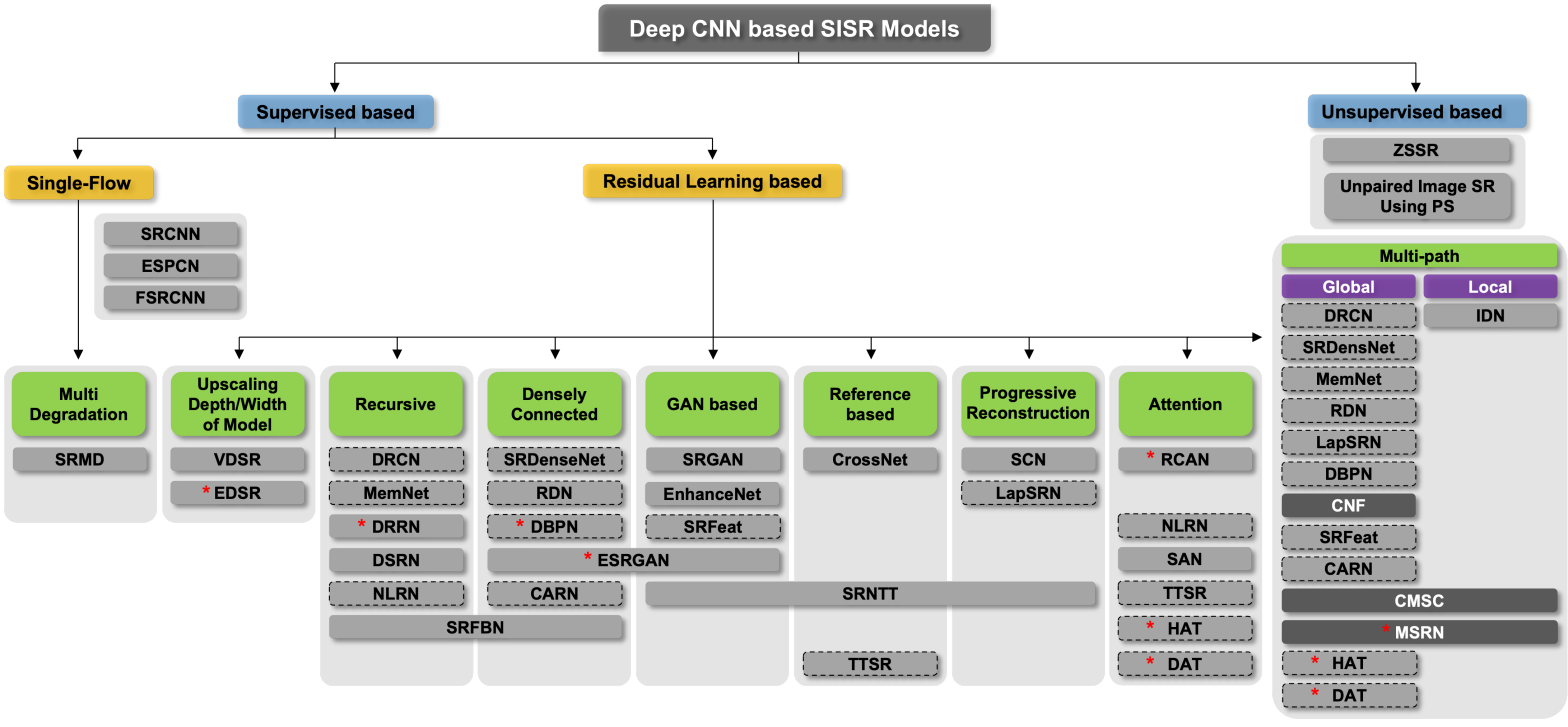
Hierarchically structured taxonomy of representative deep learning-based SR models. * indicates the model used in the experiment. The dotted line indicates a model also included in other architectural styles, and the black background is an architecture using a multi-scale receptive field.

**Figure 2:**
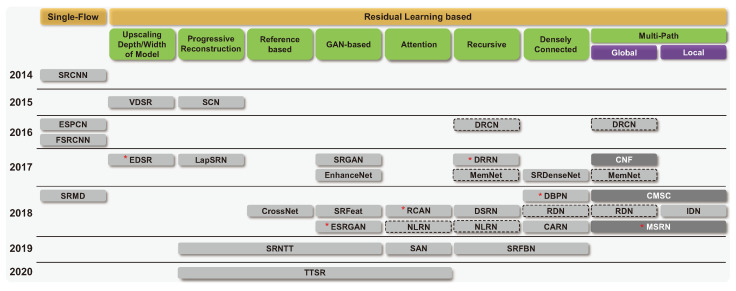
Major architectural changes in the SR model over time. * indicates the model used in the experiment.

**Figure 3:**
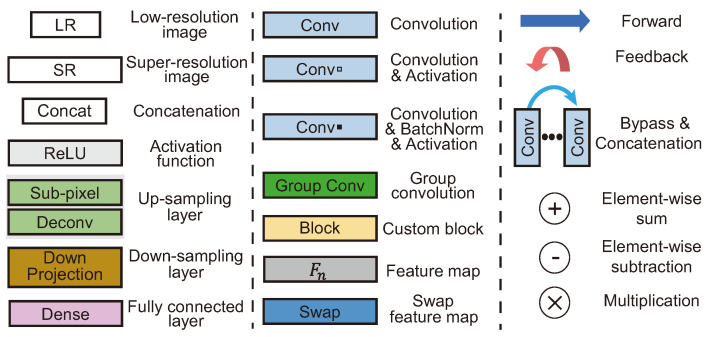
Shape compilation used for figures.

This study investigates 35 DL-based SR models and classifies them hierarchically according to their main architectural features, as shown in Figure 1. In other words, they are classified primarily based on their learning methods, i.e., supervised or unsupervised learning. Furthermore, supervised learning methods are subdivided into single-flow and residual learning architectures. Except for the initial model or a few models, residual learning has been used for most models. Residual learning is generally a structure that uses skip connections, which adds an input value of a convolution operation to an output value, preventing gradient vanishing. This structure allows for additional layers to be stacked on top of a shallow model. Skip connections, in addition to preventing vanishing gradients, play an important role in the SR task. This is due to the fact that residual learning in an SR model generally fuses the output of a CNN with the conventional interpolation method. Due to this structural advantage, the number of models that use skip connections has increased (Figure 2). In Section 4.2, we explain details for residual learning approaches. We represented the compilation of shapes used for figures, as shown in Figure 3.

### 4.1. Single-Flow Architecture

The operation direction of SR models comprises a single flow; thus, this model design is concise, which is an advantage. However, these models are difficult to deepen for high-level feature extraction as there is no skip connection or multi-path.

SRCNN [27] is the first model to apply CNN to an SR task. SRCNN is a pre-upsampling model that uses a grayscale LR image upsampled through bicubic interpolation as input. It consists of a simple network structure with three convolution layers (64 channels of a 9 × 9 kernel, 32 channels of a 5 × 5 kernel, and one channel of a 5 × 5 kernel). The patch extraction and representation layer, which is the first layer, extract patches with features from an LR input. In the nonlinear mapping layer, which is the second layer, multidimensional patches are mapped nonlinearly to other multidimensional patches. HR images are reconstructed from these multidimensional patches in the reconstruction layer, which is the final layer [27]. Mean squared error (MSE) was used as the loss function, and SRCNN was slightly better than the conventional SR method in terms of PSNR and SSIM. Attempts to stack the network deeply resulted in unstable learning and performance degradation.

**Figure 4:**
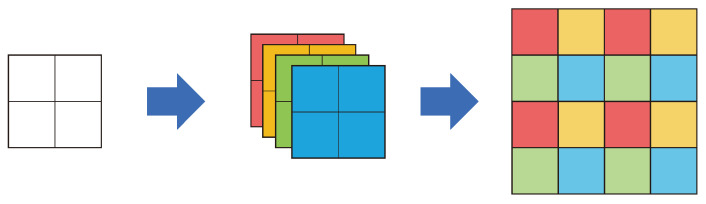
Sub-pixel convolution of ESPCN [34]. Each color represents the differences between feature map channels.

The efficient sub-pixel convolutional neural network (ESPCN) [34] is the first model that involves sub-pixel upsampling. Shi et al. [34] proposed a method for upscaling through sub-pixel convolution after extracting features from an LR image to alleviate the high computational complexity of overall SR operation. This method outputs feature maps using $n^{2}$ filters to perform ×n upscaling, as shown in Figure 4, and combines the feature maps into *n* feature maps of the upscaled scale. As sub-pixel convolution is an operation on the feature map of the LR image scale, where the computational complexity is significantly reduced, compared to the pre-upsampling method. As a result, this method was used in several subsequent post-upsampling models. However, it has the disadvantage of generating checkerboard artifacts, which are a type of noise.

The fast super-resolution convolutional neural network (FSRCNN) [33] was proposed to address the limitation that SRCNN cannot be executed in real time (24 fps). Since existing SRCNNs use bicubic interpolation to upsample input LR images, FSRCNN has a post-upsampling structure that uses transposed convolution (deconvolution) at the network’s end. Inputting LR images without preprocessing significantly increased computational efficiency. In comparison to SRCNN, FSRCNN has four additional convolutional layers, and PReLU [37] is used as the activation function instead of ReLU. The feature map is reduced in the second layer using a 1 × 1 convolution in the third layer, and then expanded again with a 1 × 1 convolution in the fourth layer. Consequently, an execution speed was increased from 1.3 fps to 24 fps or more depending on the CPU.

#### Multi-Degradation Architecture

With a multi-degradation architecture, several degradation methods have been applied to an input image to realize SR.

The super-resolution network for multiple degradation (SRMD) [38] was released in 2018. SRMD uses a single-flow structure that does not apply residual learning. Rather than using residual learning, Zhang et al. [38] used a variety of techniques, including ReLU, BN, and the Adam optimizer [39], to adequately set the depth of the model and ensure effective model training. This model is characterized by upsampling SR subimages to fit the HR scale through sub-pixel convolution. By concatenating an LR image with the corresponding degradation maps and passing them through a CNN, several SR subimages of the LR scale are generated. Degradation maps stretch each dimension of the vector, which is generated by using principal component analysis to reduce the dimension of a blur kernel vector and concatenating it with the noise-level value to match the scale of the LR image. This model approaches SR by inputting degradation information directly into the CNN.

### 4.2. Residual Learning-Based Architecture

Residual learning can be classified as a globally connected or locally connected method according to the skip connection range. The globally connected method employs interpolation to combine an upsampled LR image and the output of a model at the network’s end. This method was proposed in the VDSR model and was intended to learn residuals with HR using a CNN based on SR via the conventional interpolation method. The locally connected method uses a skip connection inside and outside the convolution block. This structure is useful for extracting high-level features because it stabilizes learning even when the models are stacked deeply, and it is commonly used in various models that adopted ResNet [40], such as SRGAN [41], and enhanced deep super-resolution (EDSR) [42].

#### 4.2.1. Upscaling Depth and Width of Model

Attempts have been made to improve performance in SR tasks by increasing network capacity. VDSR [28] and EDSR [42] are representative examples of this approach. The VDSR and EDSR models improved performance by significantly increasing network capacity when compared to existing models.

VDSR [28] is based on modified VGGNet [29] and uses a global skip connection to connect the input and output. An LR image used as input is upsampled to the HR scale through bicubic interpolation. VDSR is a ground-breaking model composed of 20 layers, which is significantly deeper layering than existing models. VDSR converges a model effectively by applying a high learning rate and gradient clipping at the start of learning.

EDSR [42] is based on a modified SRResNet [41]. In SR, an image has a fixed pixel value range; therefore, the BN layer is not required. Moreover, the use of the BN layer can degrade information in the extracted features. As a result, Lim et al. [42] did not use the BN layer in EDSR, reducing computational costs by 40%. As well as reducing computational costs, the model’s learning capacity was increased by upscaling both its width and depth [42]. The EDSR model won first place in the NTIRE 2017 challenge [43]. Lim et al. [42] demonstrated that network width and depth are strongly related to performance even in SR tasks by showing PSNR and SSIM values close to those of models released since 2018. However, there is a limit to the degree of performance improvement that can be realized by upscaling a model. Furthermore, with large-scale models, inference is slow, and the risk of overfitting is increased [44,45].

#### 4.2.2. Recursive Architecture

**Figure 5:**
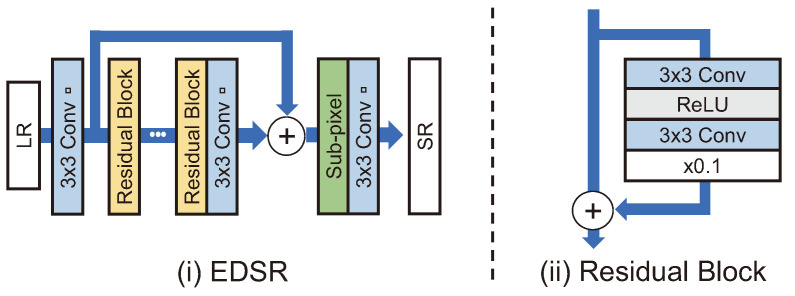
(**i**) EDSR structure [42]. (**ii**) Residual block in EDSR.

As shown in Figure 5, EDSR [42] demonstrated that expanding the depth and width of a network improved the SR performance; however, the number of parameters is significantly increased. By recursively using the same convolution layer multiple times, a recursive architecture is designed to extract higher-level features while keeping the number of parameters small.

The deeply-recursive convolutional network (DRCN) [46] extracts features using the same convolutional layer several times. To generate their respective sub-outputs, these features are connected directly to the construction layer via a skip connection. Sub-outputs are combined to derive the final output. Due to the fact that the same bias and parameters are used repeatedly, there are issues with exploding and vanishing gradients. The gradient problem was addressed by two techniques: (i) taking the average value of the features produced by the same convolution and (ii) applying a skip connection to the reconstruction layer.

**Figure 6:**
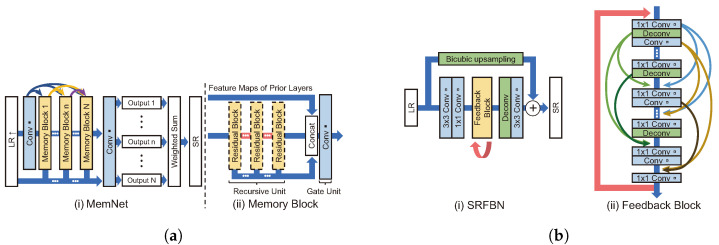
Representative models of recursive architecture. (**a**): (**i**) MemNet structure [47]. (**ii**) Memory block in MemNet. Recursive unit is the use of the same residual block multiple times. (**b**): (**i**) SRFBN structure [48]. (**ii**) Feedback block in SRFBN.

As shown in Figure 6a, the very deep persistent memory network (MemNet) [47] receives an LR image bicubic-upsampled as input. This model directly transmits input and feature maps, which are output-passed through memory blocks, to the reconstruction module. In the reconstruction module, feature maps are used to create each intermediate SR image and then fuse them to generate an SR image. MemNet convolution consists of BN, ReLU, and convolution layers in the form of pre-activation. A memory block comprises recursive and gate units, where the recursive unit is a residual block with two convolution layers. The structure of the recursive unit allows it to pass the same residual block multiple times. The feature map output from each convolution layer and the output from the memory blocks are directly connected to the gate unit (i.e., 1 × 1 convolution). The gate unit is structured to remember features that may fade away whenever they pass through a layer.

The deep recursive residual network (DRRN) [49] uses the ResNet structure as a backbone. However, the residual block is replaced by a recursive block that is used to stack several convolution layers. DRRN, unlike DRCN, recursively uses the entire block rather than a single convolution layer. To learn consistently, DRCN employs a multi-supervision strategy. Due to these structural characteristics in DRRN, the model is simplified.

The dual-state recurrent network (DSRN) [44] performs upsampling and downsampling recursively using the same transposed convolution and convolution layer. This is in contrast to DRCN and DRRN, which recursively use the same convolution layer. The concept of performing recursive upsampling and downsampling is similar to DBPN [36]. However, unlike DBPN, the process is not densely connected. Compared with DRRN, although the performance is similar at a sampling rate of ×2 and ×3, it is slightly degraded at a sampling rate of ×4, and it shows a significant difference from DBPN in that the PSNR is 1% or lower. Like DRCN, DRRN adopts a multi-supervision strategy, i.e., the final output is created by averaging all intermediate *n* outputs generated every *n* times.

The non-local recurrent network (NLRN) [50] is a DL model for estimating non-local self-similarity that was previously widely used in image restoration. Some features contain information about each image, which is referred to as self-similarity. A non-local module is used to generate feature correlation to determine self-similarity. Through 1 × 1 convolution, the non-local module extracts the correlation from each pixel in a specific area of the feature map’s neighborhood q×q. In addition, NLRN increases parameter efficiency and propagates correlations with neighboring pixels in adjacent recurrent states, taking advantage of the RNN architecture. Strong correlations for various degradations can be estimated through the inter-state flow between these feature correlations.

The super-resolution feedback network (SRFBN) [48] is a structure that operates one feedback block recursively, as shown in Figure 6b. However, similar to the DBPN [36], the outputs of each convolution in the feedback block are densely connected via recursive upsampling and downsampling. By bicubic upsampling an input LR image and adding it to the feedback block, the overall design can be considered a model that ultimately learns residuals. Although the performance for BI degradation did not differ significantly from that of EDSR [42], better performance than that of relational dependency networks (RDNs) was generally shown [24] for the complex degradation problem of BD and DN. SRFBN uses curriculum learning, which trains learning models in a meaningful order, from the easy samples to the hard ones. As a result, SRFBN may be a good fit for a complex SR degradation problem. The model for BD generated by complex degradation is specifically trained by comparing two front outputs among four outputs with Gaussian blurred HR (intermediate HR) and L1 loss three times. In addition, the model is trained by comparing two outputs at the back with the original HR. Compared to RDN, SRFBN shows better results for a complex degradation image SR problem after applying curriculum learning [48]. Note that SRFBN is constructed with parameters equivalent to 8% of those in EDSR by adopting a recursive architecture.

#### 4.2.3. Densely Connected Architecture

Feature maps from each convolution block are transmitted to the input of subsequent blocks, as in DenseNet [51]. This structure significantly reduces the number of parameters by enhancing the reuse of features and mitigating the gradient vanishing problem in object classification tasks [51]. In particular, low-dimensional features contain critical information in an SR task. This is due to the fact that even low-dimensional features can have high-frequency details (e.g., edges and textures) that must be restored in HR images [52]. Unlike ResNet’s skip connection, a densely connected architecture concatenates and uses features rather than simply adding them. This architecture ensures that important features from low to high dimensions do not vanish while passing through layers.

SRDenseNet [52] uses a post-upsampling method that employs a network in which dense blocks are applied to transposed convolution. The dense block structure connects the output of the *n*-th convolution layer from the n+1 layer to the *N* layer in a by-pass form. It can transmit the extracted feature to the bottom of the network without distorting it because the feature map generated as a result of the convolution in the dense block is used as input to the next layer via concatenation with the feature map transmitted through a by-pass.

**Figure 7:**
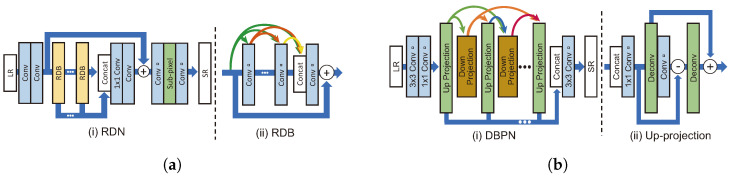
Representative models of densely connected architecture. (**a**): (**i**) RDN structure [24]. (**ii**) Residual dense block in RDN. (**b**): (**i**) DBPN structure [36]. (**ii**) Up-projection unit in DBPN.

RDN [24] was modified on the basis of SRDenseNet, and the residual dense block (RDB) was employed by adding skip connection to the dense block. The structures of the RDN and RDB are shown in Figure 7a. The RDB is designed to learn the local pattern of an LR image using all the outputs of the block immediately before reconstructing an SR image. Since the dense connection rapidly increases the number of channels, the number of channels is reduced through 1 × 1 convolution in the RDB.

DBPN [36] uses a densely connected architecture and iteratively performs upsampling and downsampling, as shown in Figure 7b. This differs from existing models that perform upsampling only once. DBPN performs upsampling twice in the up-projection unit. The progress of the up-projection unit is as follows:

(7) $$\begin{array}{cc} F_{reduct} & =Conv(1,F_{in}) \end{array}$$

(8) $$\begin{array}{cc} F_{H1} & =Deconv(F_{reduct}) \end{array}$$

(9) $$\begin{array}{cc} F_{L} & =Conv\left(F_{H1}\right)-F_{reduct} \end{array}$$

(10) $$\begin{array}{cc} F_{H2} & =Deconv\left(F_{L}\right)+F_{H1} \end{array}$$

where $F_{reduct},F_{H}$, and $F_{L}$ indicate the feature map-reduced dimensions by 1 × 1 convolution (i.e., Conv(1,x) in Equation (7)), the feature map upsampled to HR scale, and the feature map downsampled to LR scale, respectively. $F_{H1}$ can be considered an upsampling error as it differs from the original input feature map. The down-projection unit also performs downsampling twice in this structure. This process demonstrated good performance in the ×8 BI track of the NTIRE 2018 challenge [53]. However, the structure is complex, and the computational cost increases as the number of parameters increases.

The enhanced super-resolution generative adversarial network (ESRGAN) [54] is based on SRResNet. First, the BN layer is removed, as in EDSR. Second, three dense blocks (consisting of five layers of convolution with leaky ReLU) are stacked in the residual block, with the skip connections connected before and after the dense block. The residual-in-residual dense block (RRDB) is a modified architecture that is used as a GAN generator in ESRGAN.

The cascading residual network (CARN) [55] is modified through the application of group convolution and point convolution to ResNet. The existing residual block consists of convolutions and ReLU, whereas the residual-e block in CARN stacks two group convolutions and ReLU and adjusts the number of channels by a 1 × 1 convolution. By stacking residual-e blocks and a 1 × 1 convolution alternately, the cascading block densely connects the output to form a single module that comprises the network. A final network is constructed by stacking a cascading block and a 1 × 1 convolution alternately. The number of parameters is reduced by changing the existing convolution to a group convolution while using the dense connection that reuses features as much as possible.

#### 4.2.4. GAN-Based Architecture

When only the pixel-wise loss function is used in an SR task, the fine texture of a generated image tends to be blurry. A GAN-based SR model was proposed to address this problem. Adversarial learning establishes a relationship in which the generator generates an SR image and the discriminator distinguishes whether the image is real or fake. In general, it is built by adding a discriminator similar to VGGNet [29] to the existing SR model (generator). Although the images appeared to be better visually, the PSNR and SSIM values indicated deterioration.

**Figure 8:**
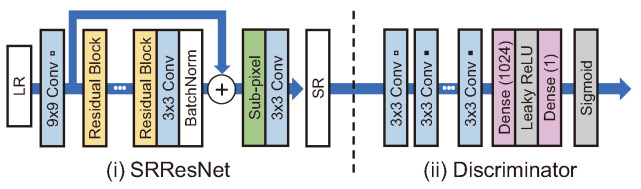
(**i**) SRResNet structure, which is a generator of SRGAN [41]. (**ii**) Discriminator of SRGAN.

SRGAN [41] is a GAN-based SR model that uses SRResNet, adopting the modified ResNet structure as a generator and a structure similar to VGGNet as a discriminator, as shown in Figure 8. The feature map-wise MSE loss was used rather than the pixel-wise MSE loss, as the existing models do not adequately represent the fine-grained texture and the SR image is blurred overall. Sub-pixel convolution is used for upsampling. The feature map-wise MSE loss calculates errors by comparing SR and HR images with the feature map obtained by passing through a pretrained VGG19 [41].

EnhanceNet [56] also grafted the feature map-wise loss onto the GAN. The difference between EnhanceNet and SRGAN is that it uses a nearest-neighbor upsampling because checkerboard artifacts are generated when using transposed convolution. The potential loss of information is prevented by applying connected residual learning globally, which adds bicubic upsampled images of input LR images.

SRfeat [57] also uses a generator that adopted the ResNet structure. A 9 × 9 filter is used for the first convolution layer, whereas the output of each residual block is compressed through 1 × 1 convolution and added through a skip connection immediately before sub-pixel upsampling. SRFeat attempted to maximize representation through a feature discriminator that uses GAN-based learning for feature maps to generate feature maps that more accurately represent actual features. Three types of loss are employed to achieve this goal: (i) the perceptual loss of the feature map-wise MSE, (ii) the image-wise GAN loss, and (iii) the feature map-wise GAN loss.

ESRGAN [54] also uses the feature map-wise MSE loss by employing VGG19. Compared to SRGAN, it is different in that feature maps are compared before passing them through the activation, which is used to show sharper edges and obtain more visually pleasing results. In addition, Wang et al. [54] proposed a network interpolation technique as follows. Given $\phi =\alpha \phi_{\mathrm{pixel}}+\left(1-\alpha \right)\phi_{\mathrm{GAN}}$ for 0<α<1, where $\phi_{\mathrm{pixel}}$ and $\phi_{\mathrm{GAN}}$ denote the parameters trained using pixel-wise loss and the GAN method, respectively. This method removes unpleasant artifacts and meaningless noise while retaining the high visual quality obtained through adversarial learning.

For super-resolution by neural texture transfer (SRNTT) [58], it is stated that the texture generated by GAN-based SR models must be a fake texture that seems real. SRNTT attempted to address this problem by grafting a reference-based method onto a GAN. The Wasserstein GAN gradient penalty [59], which measures the distance between distributions, was used as the adversarial loss and was modified based on the L1 norm to achieve more stable learning than in existing GANs.

#### 4.2.5. Reference-Based Architecture

SR is an ill-posed problem as there may be multiple corresponding HR images for a single LR image [45]. To address this issue, SR methods that makes use of similar textures in other images were proposed as a reference. Although this method can produce more visually sophisticated results, the quality of the results may vary depending on the similarity of the referenced image.

The end-to-end reference-based super-resolution network using cross-scale warping (CrossNet) [32] obtains a feature map for a similar texture by comparing the reference (Ref) image and the SR image with a flow estimator, which is a network that estimates optical flow, after generating an SR image by using an existing SR model. Slightly modified FlowNetS [60] was used as a flow estimator. The proposed flow estimator decodes a new SR image by fusing the Ref features with the features of the SR images generated from the existing SR model. EDSR [42] was used as the SR model, and U-Net [61] were used as the encoder and decoder in CrossNet, respectively. The charbonnier penalty function [62] is used as a loss function that compares SR and HR images. Although the flow estimator could be learned end-to-end, its loss was not explicitly defined.

**Figure 9:**
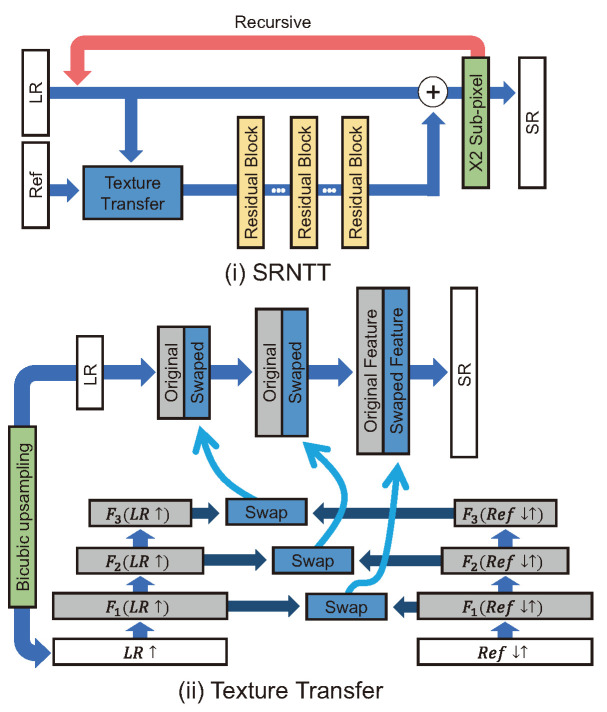
(**i**) Overall architecture of SRNTT [58]. (**ii**) The process of texture transfer using the feature map of a reference image $F_{n}\left(Ref↓↑\right)$ and an LR image $F_{n}\left(LR↑\right)$.

SRNTT [58] calculates the similarity between the LR image patch and the reference image patch through dot product by using the feature map extracted using VGGNet [29], as shown in Figure 9. Then, the feature map extracted from the LR patch is partially replaced with the feature map from the reference patch with high similarity.

(11) $$\begin{array}{c} L_{texture}=\sum_{l}\lambda_{l}||Gr\left(\phi_{l}\left(I^{SR}\right)\cdot S_{l}^{*}\right)-Gr\left(\phi_{l}\left(I^{Ref}\right)\cdot S_{l}^{*}\right){\left|\right|}_{F} \end{array}$$

The texture loss $L_{texture}$ in Equation (11) was used for texture similarity training [58]. $\left|\right|\cdot {\left|\right|}_{F}$ and ϕ denote the Frobenius norm and the feature space, respectively. Gr(·) computes the Gram matrix, and $\lambda_{l}$ is a normalization factor corresponding to the feature size of layer *l* [58]. $S_{l}^{*}$ represents a weighting map for all LR patches calculated as the best matching score [58]. Compared to CrossNet, the texture loss for the Ref image $L_{texture}$ is explicitly defined.

(12) $$\begin{array}{c} L_{total}=L_{pixel}+L_{feat}+L_{adv}+L_{texture} \end{array}$$

As shown in Equation (12), the total loss function of SRNTT consists of the pixel-wise MSE loss $L_{pixel}$, the feature map-wise loss $L_{feat}$, the adversarial loss (WGAN-GP loss) $L_{adv}$, and the texture loss $L_{texture}$. Unlike CrossNet, SR images can be created end-to-end, and textures with high similarity in the local patch are searched and imported.

The texture transformer network for image super-resolution (TTSR) [63] captures the relevance between an LR image and a reference image using Transformer architecture [64]. TTSR starts with the SRNTT model and removes all BN layers and the reference part. SRNTT employs a pretrain VGGNet [29] as a texture extractor, whereas TTSR uses a Learnable ConvNet (i.e., learnable texture extractor (LTE)) with five convolution and two pooling layers. This LTE is trained end-to-end and used to calculate the relevance (similarity) between the LR image and reference image using Q,K,V (query, key, value) attention in the feature map-wise; where Q,K,V denote the LR↑ patch feature, the Ref↓↑ patch feature, and the Ref patch feature, respectively. Also, ↑ and ↓ represent bicubic upsampling and bicubic downsampling, respectively, i.e., ↓↑ means performing downsampling and upsampling sequentially to match distributions of the Ref patch with the LR↑ patch.

The TTSR transfers the textures of patches by following this process. (i) Relevance embedding: The hard/soft attention map and similarity are calculated using the normalized inner product of the LR↑ patch feature *Q* and the Ref↓↑ patch feature *K*. (ii) Hard attention: The transferred texture features *T* are generated using hard attention by replacing the Ref↓↑ patch feature *K* with the Ref patch feature *V*. (iii) Soft attention: After concatenating *T* with the LR patch feature *F* and performing convolution on them, this is multiplied element-wise with the soft attention map *S* and added again with the LR patch feature *F* as follows:

(13) $$\begin{array}{c} F_{out}=F+Conv\left(Concat\left(F,T\right)\right)⊙S \end{array}$$

#### 4.2.6. Progressive Reconstruction Architecture

Since post-upsampling methods upsample the feature map from the end of the network to the final scale only once, they cannot extract features from the HR image space. A progressive reconstruction architecture gradually upsamples the feature map in the middle of the network to compensate for this problem.

The sparse coding-based network (SCN) [31] is a model that simulates the conventional sparse coding concept using CNN and has a structure that performs gradual upsampling. Through the patch extraction layer, the model performs sparse coding using the learned iterative shrinkage and thresholding algorithm (LISTA) [65] subnetwork, followed by HR patch recovery and a combination of the output patch. The LISTA subnetwork operates in two recurrent stages, each of which consists of two fully connected layers and an activation function that uses a specific threshold. In addition to the fully connected layer, the threshold value used for activation is learned.

**Figure 10:**
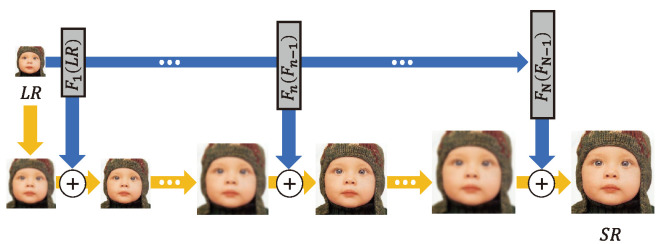
LapSRN architecture [35]. The top blue arrows represent the feature extraction branch, and the bottom yellow arrows represent the image reconstruction branch.

LapSRN [35] consists of two branches responsible for feature extraction and image reconstruction (Figure 10), respectively. LapSRN gradually upsamples an input image and extracts HR features from the image in the feature extraction branch. LapSRN is designed to enable the stable learning of a model through a residual connection between LR and HR in the image reconstruction branch. Transposed convolution is used as an upsampling method. Furthermore, because the model has several intermediate outputs, the Charbonnier loss, which is derived from the L1 loss, is used to effectively control outliers.

SRNTT and TTSR are based on progressive reconstruction to use the feature map of the reference image according to each scale.

#### 4.2.7. Multi-Path Architecture

The multi-path architecture comprises the network flows in multiple branches, and extracts features of different roles for each path. The features are transmitted and used in their original form, or they are fused. This multi-path can be categorized either as global or local.

##### Global Multi-Path

Each feature map output from multiple convolutional layers is transmitted immediately before the reconstruction layer and used together for image reconstruction. In other words, it does not rely solely on the features extracted from the final convolution layer. Due to the nature of an SR task, features that can be extracted from shallow layers have an impact on the reconstruction process.

RDN [24] connects several RDB blocks through dense connection and global skip connection. First, high-dimensional features are extracted through the mainstream, and for the local patterns to be preserved, the output of each RDB block is transmitted through a global skip connection immediately before the upsampling layer. Models constructed in this form include SRDenseNet [52], DBPN [36], CARN [55], and a multi-scale residual network (MSRN) [66]. Although SRFeat [57] also used a global multi-path, the feature map output from each block is compressed using 1 × 1 convolution and element-wise sum, which is performed through the skip connection immediately before the upsampling layer.

MemNet [47], DRCN [46], and the cascaded multi-scale cross network (CMSC) [67] generate intermediate output SR images with feature maps extracted from each block and convolution layer and weighted sum intermediate outputs to generate the final output. This is a multi-supervision strategy that can be categorized as a global multi-path because the feature maps output in each step are used as they are.

Context-wise network fusion (CNF) [68] fuses each output using several SRCNN [27] models with varying filter sizes. The roles of each unique model are used well by employing the global multi-path form, and their results can be combined adequately.

(14) $$\begin{array}{c} I_{SR}=F_{\left(S_{1},...,S_{M}\right)}\left(x\right)=\sum_{j=1}^{M}W_{j}\times S_{j}\left(x\right)+b_{j} \end{array}$$

where $I_{SR}$ denotes the final SR image, and the equation represents a method for multiplying and adding SR ($S_{1},...,S_{M}$) images of each model by the weight of the fusion layer. CNF first trains each independent model individually, then freezes all independent models learned in the previous step and trains the fusion layer. Following these steps, the CNF model is fine-tuned from beginning to end.

LapSRN [35] used the feature extraction branch and image reconstruction branch separately for each purpose, as shown in Figure 10. This can also be classified as a global multi-path form because the features extracted from each module were used unaltered.

##### Local Multi-Path

**Figure 11:**
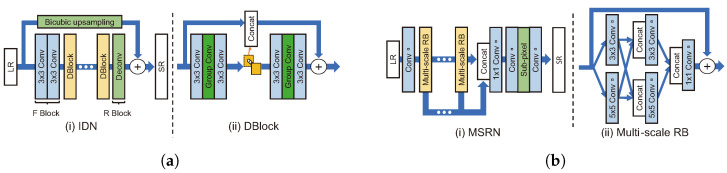
Representative models of multi-path architecture. (**a**): (**i**) IDN structure [69]. (**ii**) Distillation block in IDN. (**b**): (**i**) MSRN structure [66]. (**ii**) Multi-scale residual block in MSRN. This shows a schematic of a block consisting of multi-scale receptive fields in parallel.

This structure transmits features to multiple paths within a block. The information distillation network (IDN) [69] consists of a feature extraction block (FBlock), a distillation block (DBlock), and a reconstruction block (RBlock), as shown in Figure 11a. FBlock extracts LR image feature maps using two 3 × 3 filters. DBlock consists of an enhancement unit and a compression unit. The enhancement unit uses many local paths through a structure that divides the channel of the feature map output from the third internal convolution by $\frac{1}{n}$, concatenates $\frac{1}{n}$ with the input of the unit, and transmits the remaining $\frac{n-1}{n}$ to the next convolution layer. At the end, the concatenated feature map and the feature map extracted from the original direction are combined. Furthermore, group convolution is used in the enhancement unit’s second and fourth convolution layers to reduce computational costs and prevent an increase in the number of deep network parameters, which is why relatively few filters were used per layer. Subsequently, the output of the enhancement unit is used by reducing its dimension via a 1 × 1 convolution known as a compression unit. In RBlock, SR results are generated by upsampling the feature map extracted through transposed convolution and adding it to the bicubic upsampling LR image.

CMSC [67] used a global multi-path and constructed a stacked multi-scale cross (SMSC) module that crosses the flow of features and stacks it in a network. The multi-scale cross (MSC) module connects multiple filters of various scales in parallel and averages the inputs of the module element-wise. The averaged feature map is applied to the outputs of parallel-connected filters, each filter connected via a skip connection. The SMSC module is stacking and repeating MSC modules.

The MSRN [66] model uses both global and local multi-paths. MSRN is based on a concept similar to CMSC; however, it has three main differences from CMSC, as shown in Figure 11b. The first difference is that the proposed multi-scale residual block (MSRB) module has a relatively simple structure. The MSRB is made up of two 3 × 3, 5 × 5 convolution layers and a ReLU, followed by a 1 × 1 convolution layer, as a local multi-path structure. Second, it uses the feature map by compressing it with a 1 × 1 reduction layer rather than generating an intermediate image as the output of each block. Third, it is a post-upsampling model that uses an LR image as an input and uses sub-pixel convolution. MSRN is more efficient than CMSC, which has a pre-upsampling structure because it operates in the LR image space. In addition to these differences, CMSC adopts the multi-supervision strategy using intermediate SR results, whereas MSRN uses feature maps generated by concatenation from blocks without using BN. This is probably the reason why MSRN outperforms CMSC by approximately 1% based on PSNR.

#### 4.2.8. Multi-Scale Receptive Field

While a 3 × 3 convolution filter scale is widely used, the multi-scale receptive field architecture uses various filter sizes, such as 5 × 5 and 7 × 7.

Models that use the multi-scale receptive field structure include CNF [68], CMSC [67], and MSRN [66]. A multi-scale receptive field was applied to each SRCNN in CNF [68] using different filter sizes or layers. In addition, an MSC module with stacked filters (two 3 × 3, 3 × 3 and 5 × 5, two 5 × 5, and 3 × 3 and 7 × 7) was used in CMSC [67]. MSRN [66] operates in parallel by stacking two layers of 3 × 3 and two layers of 5 × 5 filters.

The advantage of these models is that they can take various inputs of contextual information, and the disadvantages are that the number of parameters increases as a filter larger than the commonly used 3 × 3 filters is used, and the model can be heavier because multi-scale filters are often applied in parallel.

#### 4.2.9. Attention Architecture

##### Channel Attention

Since SENet [70] using a channel attention mechanism achieved good performance in image classification tasks, various studies about channel attention have used it for SR. An LR image is primarily composed of low-frequency information. In an SR task, it is important to extract high-frequency information, such as edges and textures, required for reconstructing HR images from LR sources [71]. Of a landmark work, RCAN [71], SAN [72], and CVANet [73] effectively extracted high-frequency information through channel attention.

**Figure 12:**
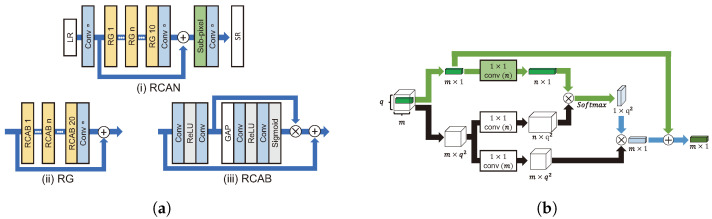
Representative models of attention architecture. (**a**): (**i**) Overall RCAN structure [71]. (**ii**) Residual group, including residual channel attention block (RCAB), in RCAN. (**iii**) RCAB. (**b**) Process of performing non-local module in NLRN [50].

As shown in Figure 12a, the residual channel attention network (RCAN) [71] grafted the channel attention onto SR using global average pooling (GAP), as in SENet. The weight of each channel is adjusted by applying GAP, ReLU, and a sigmoid function, sequentially. Subsequently, feature maps are added by a skip connection to attention weights across channels. For stable learning, residual learning is applied to both local and global spatial information. RCAN showed that channel attention was effective even for low-level vision tasks (i.e., SR) that process pixel-wise.

The second-order attention network (SAN) [72] superseded RCAN and performed channel attention as the second-order factor, which comprises GAP and covariance normalization. Second-order channel attention can reconstruct features adaptively using higher-order statistical features rather than first-order features.

##### Non-Local Attention

Self-similarity means that a region in an image is similar to another region in the same image. According to NLRN [50], the self-similarity for a specific pixel is more distributed around a proximate pixel than a distant pixel, and the correlation is extracted using a 1 × 1 convolution with a limit on the surrounding area, as shown in Figure 12b. By limiting the surrounding area, the influence on the surrounding features is increased, while the effect of noisy features is attenuated. NLRN incorporates correlations with neighboring pixels in pixel values at each position through a non-local module.

##### Transformer

TTSR [63] is proposed to use transformer architecture for the SR task. As mentioned in Section 4.2.5, TTSR applies the hard/soft attention method using (query, key, value) attention to the reference-based SR.

**Figure 13:**
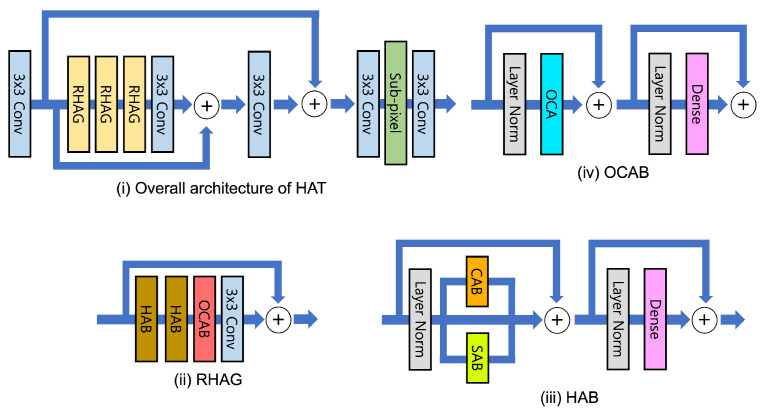
(**i**) Overall HAT structure [74]. (**ii**) Residual hybrid attention group (RHAG). (**iii**) Hybrid Attention Block(HAB) in RHAG, CAB, and SAB mean channel-attention and self-attention. (**iv**) Overlapping cross-attention block (OCAB) in RHAG.

The hybrid attention transformer (HAT) [74] addresses the limitation of traditional transformer-based models, which fail to fully exploit the architecture’s benefits due to their reliance solely on the discrete information of input pixels. As shown in Figure 13, it introduces the HAT approach that merges a channel attention-based convolution block with the self-attention mechanism inherent in existing transformer models. Leveraging this, HAT showed superior image reconstruction capabilities compared to alternative models and currently achieved the SOTA across various benchmark datasets in the SR tasks.

**Figure 14:**
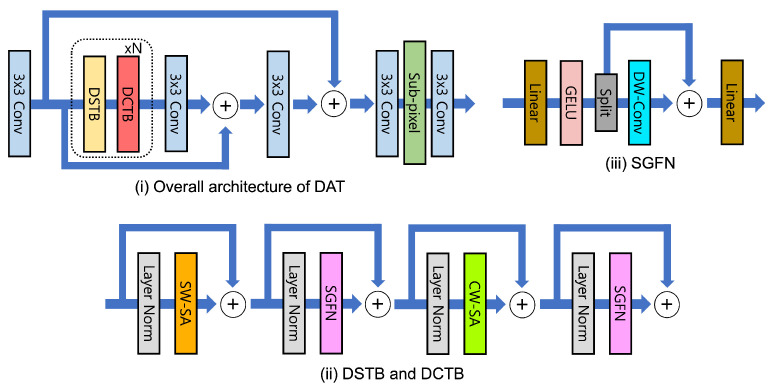
(**i**) Overall DAT structure [75]. (**ii**) Dual spatial transformer block (DSTB) and dual channel transformer block (DCTB). (**iii**) Spatial-gate feed-forward network (SGFN) in DSTB and DCTB.

The dual aggregation transformer (DAT) [75] integrates spatial and channel features both inter-block and intra-block, applying spatial self-attention and channel self-attention alternately within the transformer block to enable effective inter-block feature aggregation capturing global information, as shown in Figure 14. Furthermore, for intra-block feature aggregation, DAT introduces an adaptive interaction module (AIM) that combines the transformer’s self-attention block with a convolution block, and a spatial-gate feed-forward network (SGFN) method that separates channels in the feed-forward network and incorporates a spatial-gate to enhance the utilization of spatial information. This approach has enabled DAT to achieve outstanding performance in SR tasks.

### 4.3. Unsupervised-Based Super-Resolution Methods

DL-based SR models are trained using pairs of LR images generated by degrading HR images. Real-world images tend to have a different representation distribution from LR images generated in experiments; thus, SR for real-world images frequently performs poorly. Recently, methods to learn real images based on unsupervised learning have been proposed to address this problem.

**Figure 15:**
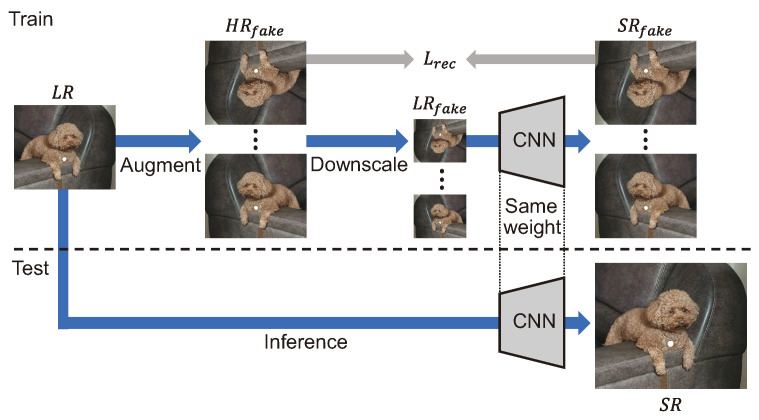
Overall zero-shot SR structure [76]. The top blue arrows represent the process of training a CNN using input images, and the bottom blue arrow represents the SR process after training.

Zero-Shot SR (ZSSR) [76] generates an LR image by degrading eight image pairs that perform flip and rotation augmentation on an input LR image. The LR image is replaced with a fake HR image in the process, as shown in Figure 15, and the relationship between the two images is trained by generating a fake LR image. Since the degradation can be specified as a hyperparameter, an SR model can be trained for various degradation conditions. However, one of the drawbacks is that the degradation must be estimated empirically based on the SR result.

**Figure 16:**
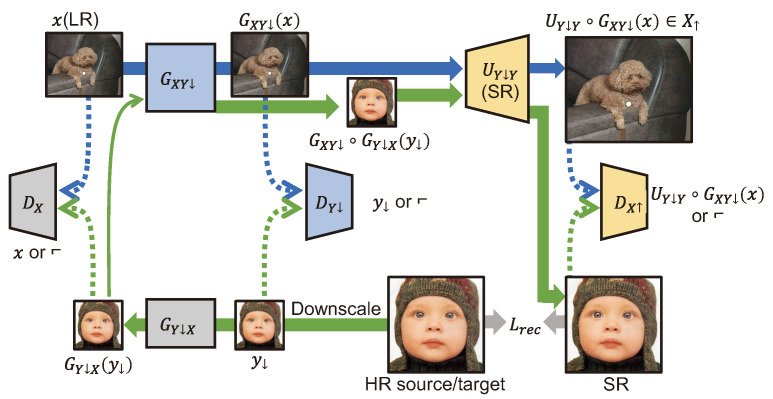
Overall structure of unpaired image super-resolution using pseudo-supervision [77]. The green arrows represent a process of learning with LR images generated from HR images, and the blue arrows represent a process of generating SR from a real image.

Maeda et al. [77] proposed unpaired image super-resolution using pseudo-supervision for learning SR by targeting real images with three GAN models. The overall structure is shown in Figure 16. $G_{Y↓X}$ learns to generate an LR image that has a distribution similar to the distribution of the real image from HR images, and $G_{XY↓}$ converts the distribution of the real image into the image distribution learned by an SR model. RCAN [71], which learns to generate a texture that replicates the distribution of the real image in a GAN form, was used as the $U_{Y↓Y}$ model. To perform SR well, it transfers the domain from the real image distribution to the image distribution learned by the SR model.

## 5. Object Detection Works Briefing

**Figure 17:**
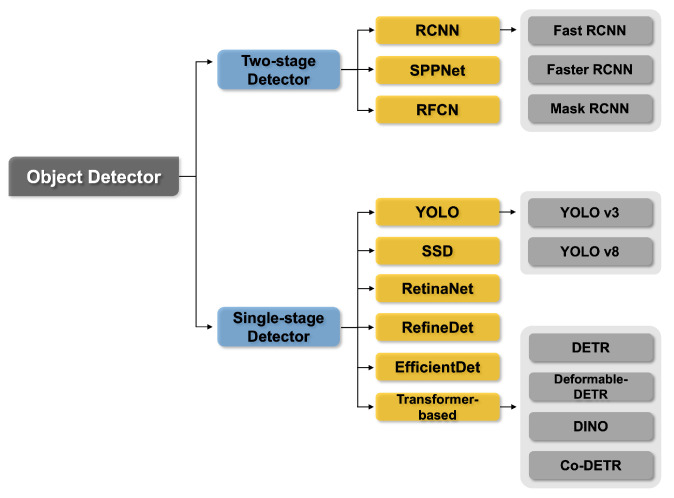
Tree of object detection models. OD models can be classified into two-stage and single-stage frameworks.

As shown in Figure 17, OD models are primarily classified into two-stage and single-stage methods. The two-stage method involves searching for regions where an object is likely to exist, either through selective search or by using a region proposal network (RPN), and then classifying the object for the corresponding regions. The single-stage method searches for regions where an object will probably be and classifies the object for the searched regions at the same time. In general, single-stage methods outperform two-stage methods in terms of network inference speed, whereas two-stage methods outperform single-stage methods in terms of accuracy. Recently, a transformer-based OD model has been proposed as a single-stage approach that outperforms two-stage detectors and achieves state-of-the-art performance on various benchmark datasets.

### 5.1. Two-Stage Network

Representative two-stage network technologies include R-CNN [78], Faster R-CNN [6], and Mask R-CNN [79].

R-CNN [78] is an initial two-stage network based on DL in which region proposal, feature extraction, and classification are all performed in separate models. First, thousands of potential candidate object regions are extracted using a selective search algorithm [80], and each proposed region is then passed through a CNN for feature extraction. An SVM is applied to the extracted features to classify objects. This is a bottleneck process because each proposal’s inference is performed separately.

SPPNet [81] employed a spatial pyramid pooling (SPP) structure to accelerate feature extraction for images or subimages of an arbitrary size/scale, which are bottlenecks in R-CNN. SPP is a method for classifying feature maps into multi-scale predefined bins from which output features of the same size can be generated regardless of input sizes. SPPNet projects a region of interest (RoI) onto a feature map and feeds it to the SPP structure for CNN features to be extracted for all RoIs with a single inference. R-CNN is inefficient because it performs *N* inferences after generating cropped and warped patches to fit the *N* RoIs generated by the region proposal method to the CNN input. In the Fast R-CNN, the SPP structure is then devolved into a structure known as the RoI pooling layer. SPPNet has several drawbacks as it uses region proposals and a separate SVM classifier.

Faster R-CNN [6] improved the overall processing speed and detection performance using an RPN by replacing the existing region proposal method, which was a bottleneck in R-CNN and Fast R-CNN. The previously used selective search method performs super-pixel-based segmentation and merges various super-pixels to derive bounding boxes around a region as output. Due to the fact that it incurs enormous computational costs and does not take into account the semantics of an input image, this method increases the risk of false positives. Conversely, detection performance is maintained even when the number of proposal candidates is small because the RPN learns the position where the object is likely to be based on the position of the target object. In addition, speed is enhanced as the feature extractor and backbone are shared. Faster R-CNN detects objects faster than the existing R-CNN or Fast R-CNN because it is designed to perform end-to-end detection from the proposal stage by attaching a box regression head and a classification loss head to the network’s end. However, there are some limitations in applying it to real-time video.

The region-based fully convolutional network (RFCN) [82] is a model that replaced the RoI pooling and FC layers of Faster R-CNN with an FCN structure to preserve the RoI positional information. As an FCN output, a position-sensitive score map is generated. Box regression and classification heads are attached to the FCN, allowing for detection to be performed without additional structures. RFCN has the advantage of being faster and have more consistent performance as it uses the FCN structure.

Mask R-CNN [79] involves a simple method for adding a mask prediction head in a parallel structure to the basic structure of Faster R-CNN, enabling OD in pixel units. The mask prediction head is composed of a simple fully convolutional structure. The mask prediction head does not increase computational costs significantly because the backbone network weight is shared between the box regression head and classification head. Recently, Mask R-CNN has been applied in various studies owing to its outstanding detection performance and high detection speed. In addition, Mask R-CNN has shown enhanced performance compared to its initial release by being fused with the feature pyramid network [83], focal loss [84], and GIoU loss [85].

### 5.2. Single-Stage Network

Representative examples of single-stage networks include YOLO [7], RetinaNet [84], RefineDet [86], and EfficientDet [4].

YOLO [7] is the most used OD method in real-world applications, such as autonomous driving or face detection. YOLO detects objects by selecting the highest score through the extraction of bounding boxes for each cell and the score for a class after dividing the receptive field into 7 × 7 grid cells. YOLO has a single neural network structure, but its processing speed is extremely fast because all layers are composed of convolutions. However, its accuracy is slightly lower than those in two-stage-based methods because YOLO cannot effectively detect a small target object or an image that contains cluttered background as only one class can be predicted in one cell. Advanced versions of YOLO, i.e., YOLOv2 [87] and YOLOv3 [88], have been developed to overcome these limitations. The latest version of YOLO uses CSPNet [89] as a backbone to reduce inference cost while increasing input resolution and applies various augmentation techniques when training the model to efficiently improve detection performance for small objects [90].

SSD [91] is a model that detects objects by predicting the box regression value and class score for each multi-scale feature map generated from a CNN model and then collects them through non-maximum suppression (NMS). A separate complex structure is not required as the multi-scale features are feature maps generated after performing convolution, and SSD has the advantage of rapidly capturing small-to-large objects because detection is directly performed on feature maps of various scales. SSD has influenced many subsequent studies with its simple network structure and outstanding detection performance.

RetinaNet [84] uses the following focal loss to address the class imbalance problem that occurs because negative samples are commonly found when training a detection model.

(15) $$\begin{array}{c} Focal\;Loss\left(p_{t}\right)=-{\left(1-p_{t}\right)}^{\gamma }\log\left(p_{t}\right) \end{array}$$

where $p_{t}$ denotes the prediction score and γ denotes the hyperparameter for focusing. In Equation (15), ${\left(1-p_{t}\right)}^{\gamma }$ is considered a scaling factor that reduces the contribution of samples that can be easily classified in the learning process and weights the samples that are difficult to classify. It has a single-stage structure that calculates the box and class label after extracting the multi-scale feature map based on FPNs. Consequently, RetinaNet achieves high detection performance and processing speed due to its simple structure and focal loss function. In particular, the proposed focal loss function has been applied to various detectors because it is simple to use and effective.

RefineDet [86] is a one-stage detector that takes satisfactory advantage of two-stage detectors. RefineDet consists of an anchor refinement module (ARM) that generates refined anchors and an OD module (ODM) that performs detection and classification. The ARM generates an anchor for each cell and determines whether each anchor is in the foreground after dividing an input image into cells, as in YOLO. Furthermore, the ARM simultaneously adjusts the position of the anchors. Filtered anchors are fed into the subsequent ODM and used to perform bounding box regression and classification. Therefore, RefineDet’s detection performance is superior to that of other existing one-stage detectors because it performs two-step cascaded regression. Although its appearance is similar to that of a two-stage detector, it does not require a separate model for region proposals, as in the R-CNN series, and it has a processing speed advantage because inference is performed on a single network.

EfficientDet [4] is a computationally efficient detection model that comprises a weighted bidirectional feature pyramid network (BiFPN) that effectively extracts multi-scale features. EfficientDet’s BiFPN removes unnecessary edges to improve an FPN’s computational efficiency and takes top–down and bottom–up processes into account to improve accuracy. In addition, a fast normalized fusion method was proposed to consider weightings during the fusion of each layer of an FPN by emphasizing that the contribution of each scale to the output feature map must vary when differently scaled feature maps are fused. Compound scaling, which considers factors that determine the size and computational cost of a model, such as input depth, width, and resolution, was introduced and applied to the EfficientDet model by experimentally finding the optimal coefficient.

#### Transformer-Based Network

Transformer [64] is an encoder–decoder model based on attention, which was initially proposed for sequential data processing such as natural language processing and has shown excellent performance. Several attempts have recently been made to apply the transformer to the image domain. In the field of image classification, models such as ViT [92] and Deit [93] divide the image into grid-type patches and sequentially input them to the transformer have achieved SOTA. DETR [94] and Deformable DETR [95] demonstrate the potential of transformer-based models in the field of OD.

DETR [94] is a single-stage detector that uses self-attention to perform OD. The study [94] emphasizes the importance of a post-processing method, such as NMS, because existing detectors typically have an anchor structure that collapses near-duplicate predictions for one target object. To address this issue, a transformer-based model, DETR, that learns how to match target objects and predictions one-to-one, was proposed. In DETR, RoI candidates are searched for using predefined image embedding features called *N* image queries rather than anchors. Image queries do not make redundant predictions due to Hungarian matching. Training a model based on the transformer can be time-consuming, the processing speed is not slower than that of Faster R-CNN, and its detection performance is also good. However, it has been reported that the detection performance is significantly degraded when the target is small, as the receptive field is limited by its structural characteristics.

DINO [96] addresses the performance degradation issue in detecting small objects, previously encountered by DETR [94], through contrastive denoising training and mixed query selection approaches. The contrastive denoising training involves training with a total of 2N queries, both positive and negative, for a single ground truth bounding box. Each query is augmented with different levels of noise, with the bounding box having lesser noise represented as a positive query, and the other as a negative query. This approach allows for the positive query to predict the actual ground truth bounding box, while the negative query is guided to predict the "no object" background, thereby overcoming the traditional issue of performance degradation in scenarios with small or overlapping objects. Mixed query selection combines the static anchor and content query approach of the original DETR [94] with the dynamic anchor and content query method from Deformable DETR [95], utilizing dynamic anchors and static content queries. This fusion enables the model to leverage better positional information for extracting more comprehensive content features from the encoder, thus enhancing performance.

Co-DETR [97] significantly advances the performance and computational efficiency of DETR-based detectors by facilitating more effective training. It identifies that the one-to-one set matching strategy employed by DETR-based detectors underperforms compared to the one-to-many label assignment approach used in traditional object detection models such as Faster R-CNN [6] and RetinaNet [84]. To address this, Co-DETR enhances encoder supervision by integrating a versatile auxiliary head, employing a collaborative hybrid assignments training method. This method generates customized positive queries based on label assignment for each auxiliary head, improving detector performance while maintaining the benefits of end-to-end training. Applied to state-of-the-art DETR-based detectors, including Deformable-DETR [95], DAB-DETR [98], and DINO [96], this approach now demonstrates SOTA performance across various OD benchmark datasets.

## 6. Experiment

Each representative SR model was selected from the architecture categories classified in Section 4 to experiment on the change in OD performance according to the various SR model’s architectures. Note that the reference-based architecture was excluded from the experiment because its performance varies depending on the similarity between reference and LR images.

The experimental process is described as follows. (i) The OD datasets are degraded with ×2 and ×4 reduction coefficients via BI, BD, and DN methods. (ii) The degraded datasets are restored using each SR model. (iii) The performance of the object detectors is measured based on the dataset generated by each SR model. Note that we trained detectors for the Widerface dataset [13], and used the pretrained detectors for the MS COCO dataset [3].

### 6.1. Datasets

The publicly available MS COCO [3] and Widerface [13] datasets were used for general OD and face detection in the experiment, respectively. These datasets contain image files in JPEG format, but the SR models provided by the authors were trained with PNG format images. As a result, using those models directly in the experiment will significantly degrade performance. The difference in compression type between JPEG and PNG image formats is responsible for this phenomenon. Due to the fact that JPEG is a lossy compression format and PNG is a lossless compression format, the representation distributions of the training data and test data differ. Thus, we converted the DIV2K [99] dataset from PNG format to JPEG format, then retrained all SR models on the JPEG version of the DIV2K. Furthermore, the MS COCO dataset categorizes objects to be detected into small, medium, and large based on their size. Similarly, the WiderFace dataset classifies objects into easy, medium, and hard based on the detection rate. Therefore, our experiment considers a variety of object types and sizes.

### 6.2. Training Details

To experiment in the same environment, we train the SR model first and then evaluate the performance of the pretrained OD model using the outputs of SR models. Thus, images generated by the SR model must be in an input format suitable for the OD model in terms of image channels. Although the recently proposed SR models primarily receive three RGB channels in the RGB color space and output three RGB channels, occasionally a model receives the luminance channel of the YCbCr color space and outputs one channel, e.g., DRRN [49] and MSRN [66]. Thus, we modified the input and output channels of the models to three channels.

The main training information for each model is summarized in Table 1 (The codes are available at https://github.com/dnap512/SROD (accessed on 15 April 2024)). We train the SR models on the DIV2K training set for 300 epochs, with an Adam [39] optimizer using L1 loss. The initial learning rate is set to 1 × $10^{-4}$, and it drops by a factor of 0.5 after 200 epochs. We use MSE loss to train DRRN because DRRN trained with MSE loss outperformed DRRN trained with L1 loss. Since MSE convergence is slower than that of L1 loss, the DRRN model is trained for 1000 epochs; the learning rate decreases by a factor of 0.5 after 500 epochs. The batch size and patch size are set as suggested by the authors of each model, and flip and rotation augmentations are applied. ESRGANs are trained in two steps. In the first step, the generator RRDB is fine-tuned for 250,000 iterations at an initial learning rate of 2 × $10^{-4}$ using only the L1 loss from the BI pretrained model provided by the authors. Second, the RRDB trained in step 1 is used as a generator and trained for 400,000 iterations with GAN loss as a discriminator. In Table 1, RRDB denotes the RRDB trained in step 1.

For the transformer-based SR Models, HAT and DAT, given their larger number of parameters compared to traditional CNN-based SR models, training is conducted over 1000 epochs utilizing the Adam optimizer with L1 loss. The learning rate is set at 2 × $10^{-4}$, and an exponential moving average gradient decent is additionally employed to enhance the training process.

### 6.3. Results and Analysis

#### 6.3.1. Analysis Summary

(i) If only the SR output format and OD input format are compatible, OD performance will improves by super-resolving LR images without joint training. (ii) OD performance improves proportionally to the PSNR and SSIM metrics of the SR model. (iii) Analysis with model architectures: (a) SR model trained with GAN architecture significantly improves OD performance even though PSNR and SSIM are low. (b) The performance improvement rate of the transformer-based OD model is the highest. (iv) OD performance improves particularly high for small objects on average. (v) For the latest DETR-based OD models, due to their commendable performance even at the baseline, they exhibit a relatively modest rate of performance improvement.

#### 6.3.2. MS COCO Result

**Figure 18:**
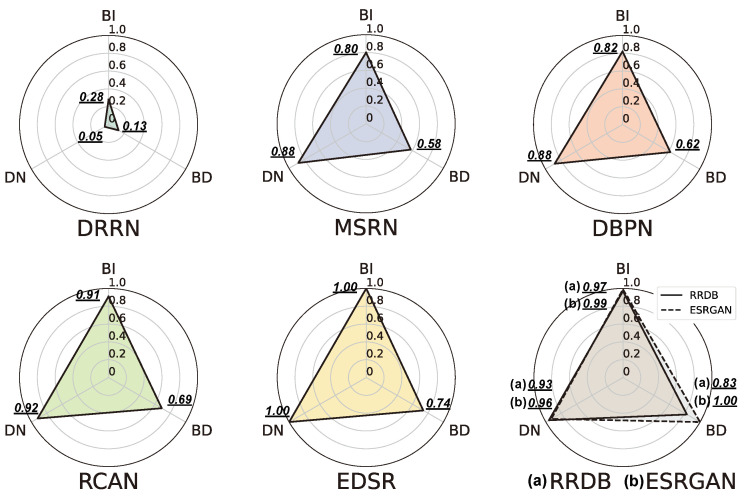
Relative OD enhancing index of each SR model for each degradation method (i.e., BI, BD, and DN). Given $\frac{\Delta P_{\mathrm{SR};d}}{\Delta P_{max(\mathrm{SR});d}}$ for d={BI,BD,DN} and SR={DRRN,MSRN,DBPN,RCAN,EDSR,RRDB,ESRGAN}, we compute the relative OD enhancing index. (Dataset: MS COCO 2017 validation set [3]).

The experimental results (i.e., mAP) obtained on the MS COCO dataset are summarized in Appendix A Figure A8. Also, we calculate the performance enhancement rate ΔP of the target compared to the baseline, and visualize it in Figure 18.

The baseline means the OD result for an image upsampled with the bicubic upsampling method from the LR image. The performance enhancement rate between the baseline and the OD result of the data generated using SR models is calculated as follows:

(16) $$\begin{array}{c} \Delta P=(\frac{mAP_{SR}}{mAP_{Baseline}}-1)\times 100 \end{array}$$

where $mAP_{SR}$ and $mAP_{Baseline}$ denote the mAP performance of the detector for the SR data and the baseline results, respectively.

As shown in Appendix A Figure A8, OD performance improves significantly in most conditions when compared to bicubic interpolation. However, in the case of HAT and DAT for BI, despite achieving the highest PSNR, their performance declined compared to the baseline on the COCO dataset. The OD model with pretrained weight is used in this experiment, and only the SR model is newly trained. This means that, if only the SR output format and OD input format are compatible, OD performance will improve by super-resolving LR images without joint training.

#### 6.3.3. Widerface Result

The overall experimental results of the Widerface dataset are summarized in Appendix A Figure A9, and the performance enhancement rate is obtained by experimenting similarly as on MS COCO. Notably, OD performance on Widerface is significantly higher than for MS COCO when bicubic interpolation (i.e., baseline) is used.

#### 6.3.4. Enhancing Detection Using Each SR Model

##### MS COCO

The average enhancement rate of OD performance of each SR and OD model is shown in Table 2. Note that the rate of DN performance enhancement is generally higher than that of BI and BD. Since the denoising effect of the compared SR models is superior to the DN baseline (i.e., bicubic interpolation), the detection performance of the subsequent OD model appears to be affected.

The enhancement rate of object detection performance of each SR model for the MS COCO dataset is shown in Table 3. Generally, the performance enhancement rate increases when the PSNR value of an SR model is high. However, there are exceptions; for HAT and DAT, while achieving high PSNR in BI, they exhibit lower performance compared to the baseline on MS COCO datasets, e.g., EDSR, which recorded a relatively lower PSNR value and showed better performance than that of RRDB in BI and DN. Also, ESRGAN outperformed all other models when it came to BI and BD performance enhancement rates.It appears that representing textures in greater detail by applying adversarial learning improved performance. The DN performance enhancement rate of ESRGAN is less than that of RRDB or EDSR, due to unpleasant artifacts in SR images and the inability to adequately remove noise compared to EDSR, as shown in Appendix A Figure A3.

**Figure 19:**
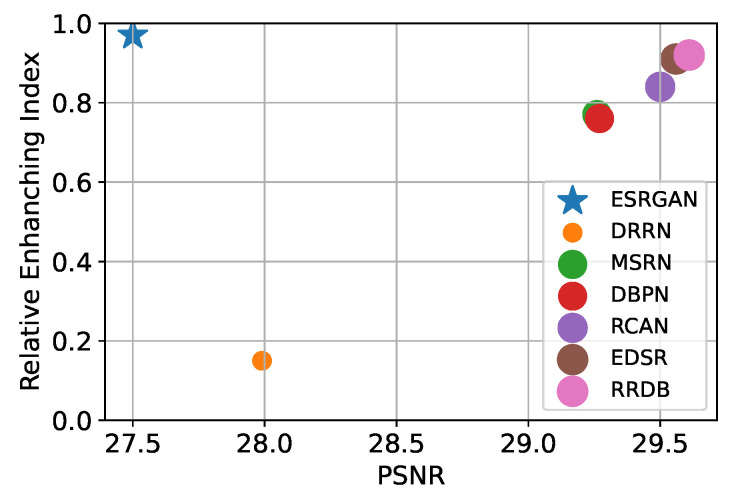
Relative OD-enhancing index per PSNR index for each SR model. Note that ESRGAN [54] is a model that trained RRDB [54] backbone with adversarial learning. With the adversarial loss, a higher enhancement rate for OD can be obtained even if the PSNR indicator is low. (Dataset for OD: COCO 2017 validation set [3], Dataset for PSNR: Set5 [101] ×4).

**Figure A1:**
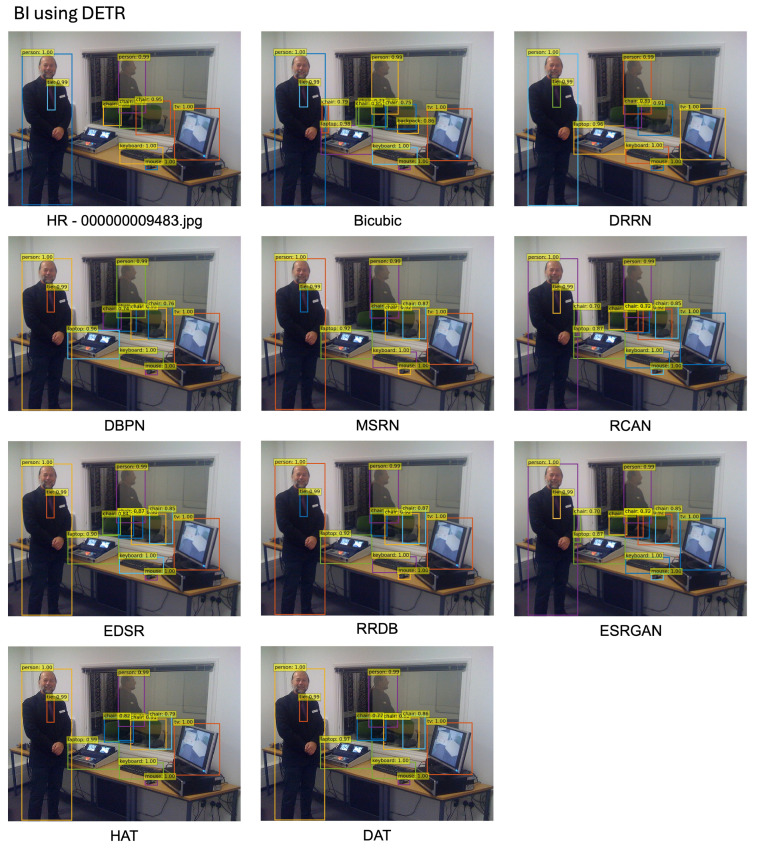
Examples of inference by applying DETR [94] to SR images. SR images are the result of applying each SR method to the LR COCO dataset [3] degraded by **BI**.

**Figure A2:**
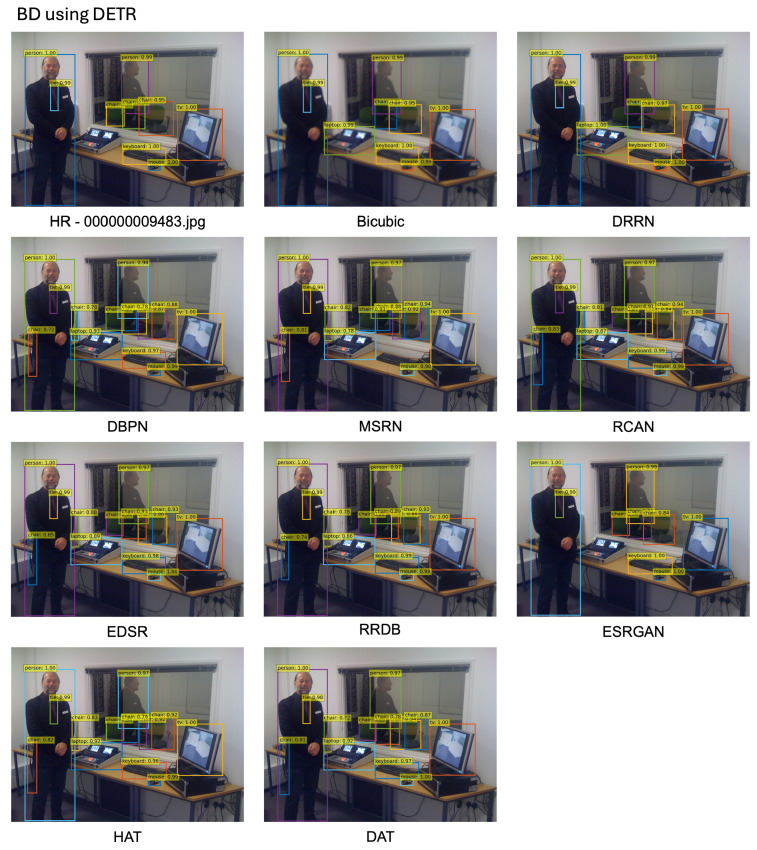
Examples of inference by applying DETR [94] to SR images. SR images are the result of applying each SR method to the LR COCO dataset [3] degraded by **BD**.

**Figure A3:**
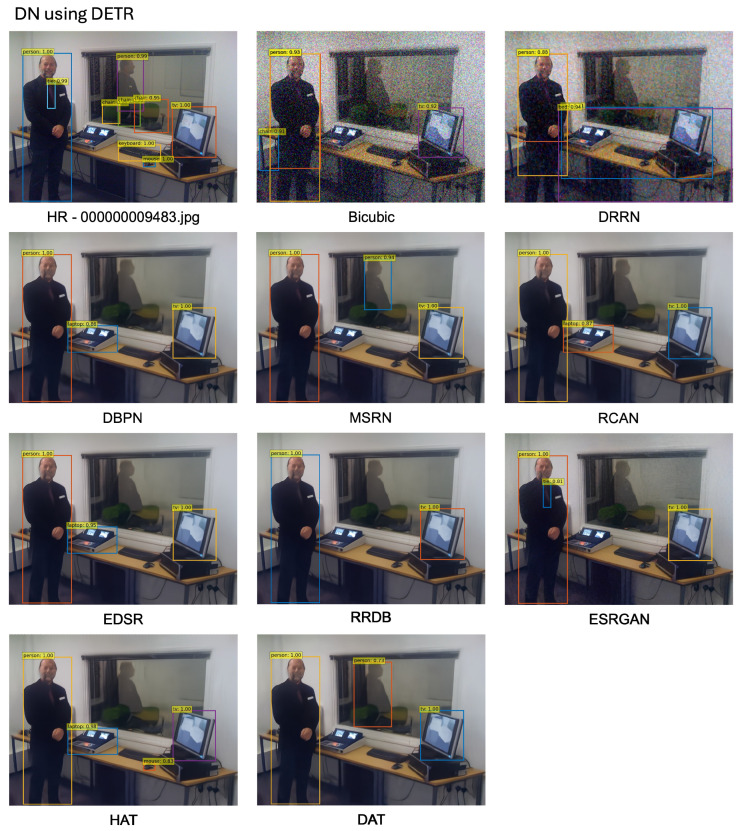
Examples of inference by applying DETR [94] to SR images. SR images are the result of applying each SR method to the LR COCO dataset [3] degraded by **DN**.

**Figure A4:**
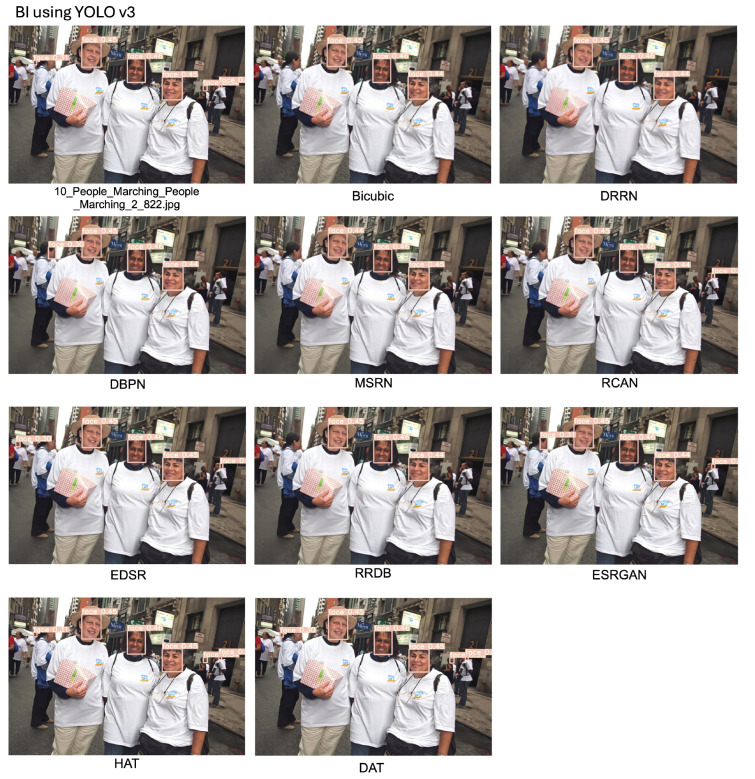
Examples of inference by applying YOLOv3 [88] to SR images. SR images are the result of applying each SR method to the LR COCO dataset [3] degraded by **BI**.

**Figure A5:**
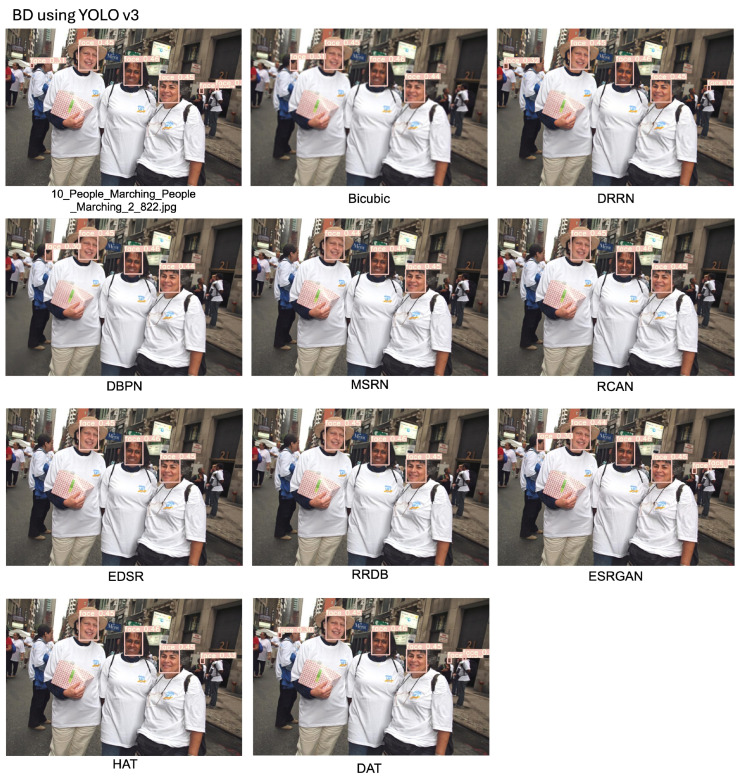
Examples of inference by applying YOLOv3 [88] to SR images. SR images are the result of applying each SR method to the LR COCO dataset [3] degraded by **BD**.

**Figure A6:**
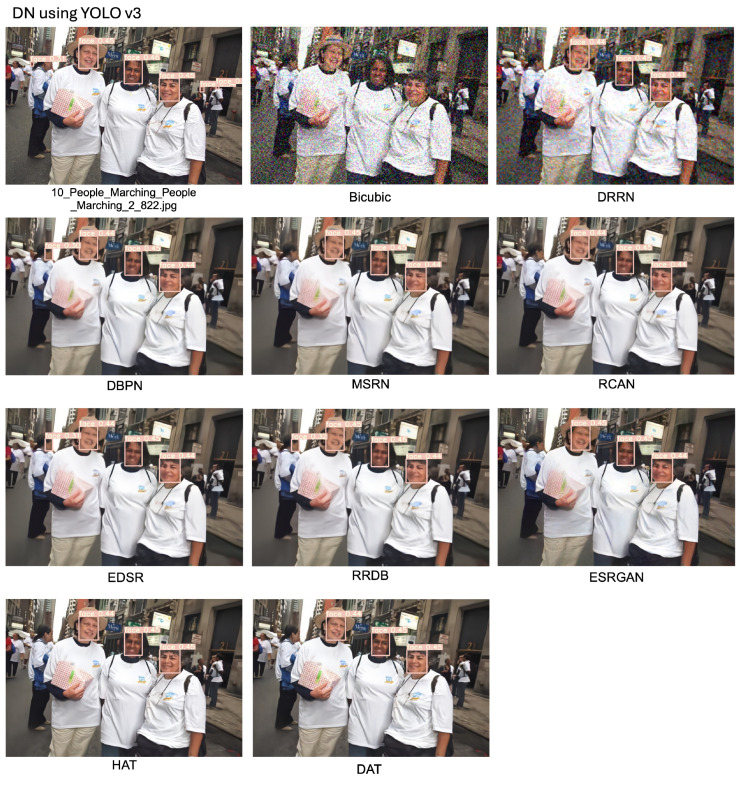
Examples of inference by applying YOLOv3 [88] to SR images. SR images are the result of applying each SR method to the LR COCO dataset [3] degraded by **DN**.

The ranking regarding the total average enhancement rate of the detection performance is equal to the DN ranking in Table 2, because the DN value is too high. For a fair comparison, we averaged each degradation enhancement rate using min-max normalization to adjust the scale. In the case of HAT and DAT, since the performance enhancement rate in BI decreased, the relative index was not indicated. Consequently, ESRGAN achieved the highest enhancement rate of 0.95. Although RRDB and ESRGAN have the same model structure, the latter showed a higher enhancement rate. This indicates that adversarial learning improves detection performance. Figure 19 presents a graph showing the PSNR value of each SR model and the normalized value of the OD performance enhancement rate. The trend is roughly proportional to the PSNR index (excluding ESRGAN; marked star), confirming that a higher PSNR index enhances OD performance in pixel-wise training. As shown in Appendix A Figure A1, Figure A2, Figure A3, Figure A4, Figure A5 and Figure A6, even though the SR models trained with adversarial and content loss achieve lower PSNR compared to those trained only with pixel loss, they bring significant gains in perceptual quality [45,56,100]. Furthermore, with adversarial loss, a higher enhancement rate for OD can be obtained even if the PSNR indicator is low.

##### Widerface

**Figure A7:**
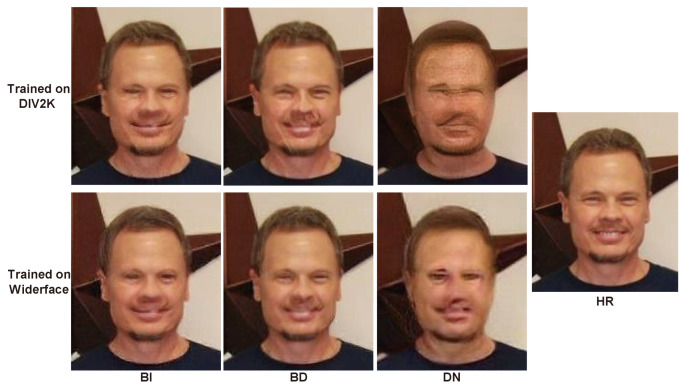
The result performing ×4 SR on the image of the ‘13_Interview_Interview_ On_Location_13_334.jpg’ image of the Wider Face validation set [13] through ESRGAN [54] trained on Widerface [13] and DIV2K [43], respectively.

**Figure A8:**
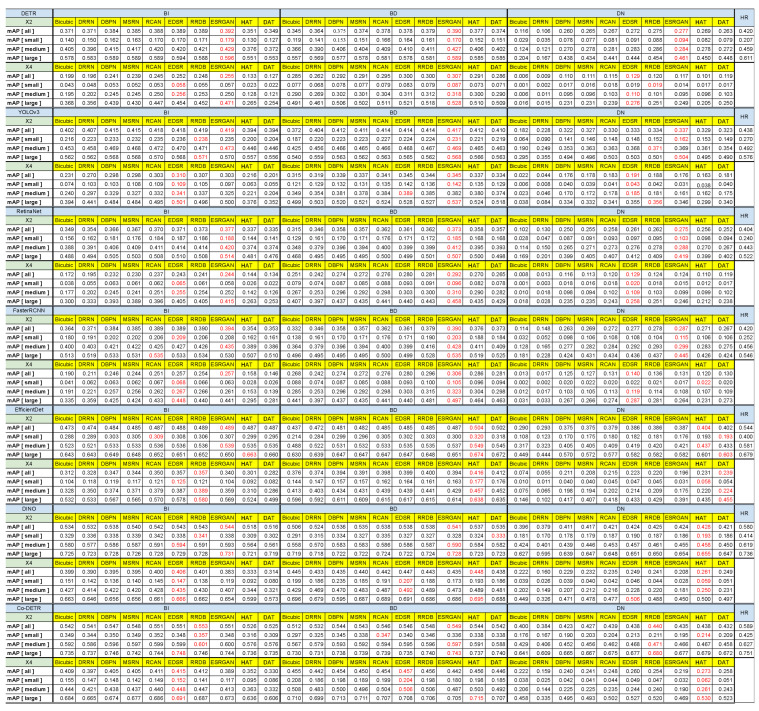
Object detection performance for COCO val2017 [3] of YOLOv3 [88], EfficientDet [4], RetinaNet [84], Faster R-CNN [6], DETR [94], DINO [96], and Co-DETR [97] according to each SR model.

**Figure A9:**
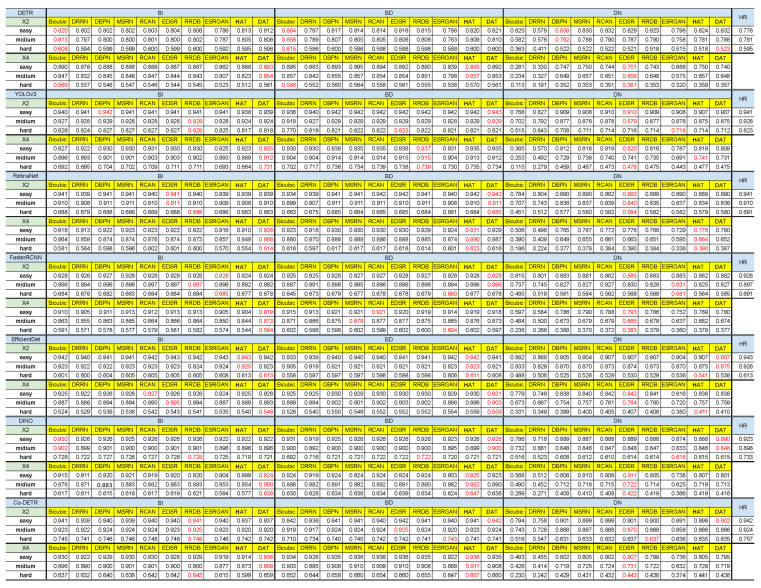
Object detection performance for the Wider Face validation set of YOLOv3 [88], EfficientDet [4], RetinaNet [84], Faster R-CNN [6], DETR [94], DINO [96], and Co-DETR [97] according to each SR model.

The performance enhancement rate in Table 4 demonstrates a trend similar to the PSNR index of the SR model. In BI and BD, DRRN and ESRGAN perform worse than the baseline. Additionally, for HAT, while recording the worst performance in BI, it achieves the best performance in BD and the second-best performance in DN. Although DRRN has the lowest performance enhancement rate on the MS COCO dataset, it also has the lowest performance enhancement rate on the Widerface dataset. ESRGAN has a negative value on Widerface despite having the highest enhancement rate for BI and BD on MS COCO. Note that the inability to represent the facial features properly and the unpleasant artifacts (Appendix A Figure A7) are the reasons for the performance degradation. Overall, the extremely high DN performance enhancement rate is observed on the MS COCO dataset because the SR model eliminated the Gaussian noise. The SR model fails to significantly improve the performance rate of the OD detector when detecting faces. As shown in the sample images of Appendix A Figure A4, Figure A5 and Figure A6, the SR model frequently destroys the texture of facial features, which appears to have affected face detection. The proportion of human faces in the DIV2K training dataset is low, and it appears that face-related features are not learned sufficiently, thereby causing faulty restoration.

Thus, we further experimented with RRDB and ESRGAN by sampling 2400 images from the Widerface training set. Table 5 shows the average comparative performances of all detectors on the Widerface validation hard set, and sample images for Table 5 are shown in Appendix A Figure A7. The performance of ESRGAN decreases compared to RRDB when training with DIV2K, however ESRGAN improves compared to that of RRDB when training with the Wideface dataset. Thus, according to Table 5 results, we confirm that training an SR model using adversarial learning can have a positive effect on face detection performance if the features of objects are sufficiently trained.

#### 6.3.5. Performance Improvement of Object Detector

##### MS COCO

The performance enhancement rate of OD models for each SR is shown in Table 3, where AP-all is the enhancement rate for all objects, and AP-small is the performance enhancement rate for small objects. As shown in Table 3, AP-small is higher than AP-all on average. Small objects could not be adequately detected in images with poor quality or noise in OD; however, SR has proven to be a solution. DETR has the highest average performance enhancement rate for each SR model, and RetinaNet exhibited the highest performance enhancement rate for detecting small objects.

Overall, the performance enhancement rate of the detectors tends to be higher when the detector’s performance is lower. We compute the performance maintenance rate of each detector for the baseline as follows:

(17) $$\begin{array}{c} P_{degradation}=\frac{mAP_{Bicubic\;image}}{mAP_{Original\;image}}\times 100 \end{array}$$

The detailed figures on MS COCO are shown in Table 6, which is similar to the reverse order of performance enhancement, except for DETR, which has a transformer architecture. Therefore, it can be inferred that detection performance can be significantly enhanced without changing the structure of the OD model, even with a detector of relatively low performance, if SR is used to increase the input image quality.

In particular, the performance enhancement rate of DRRN seems to be significantly lower than those of other SR models, and this difference appears to depend on the location and method of upsampling. DRRN is a recursive architecture with a pre-upsampling model that uses the bicubic upsampling result for LR as input. Therefore, performance is reduced due to the significant influences of bicubic interpolation used for more complex degradation methods, such as BD and DN. As a result, the post-upsampling structure enhances the performance of the detector via SR, and performing upsampling in the model as an upsampling method, e.g., transposed convolution or sub-pixel, results in a higher performance enhancement rate.

##### Widerface

The average performance enhancement rates for all SR models for each detector are shown in Table 7. The performance enhancement rate for small objects is higher in most cases (similar to the MS COCO results), and YOLOv3 achieved the highest average performance enhancement rate, while EfficientDet achieved the lowest. The enhancement rate of the medium set is greater than that of the hard set in the EfficientDet case, which appears to be due to incomplete learning about face detection considering that the enhancement rate increased as the object became smaller in the MS COCO dataset. The overall enhancement rate in Widerface is not that large compared to that in the MS COCO. There was no significant change in BI, and it showed a relatively high enhancement rate due to the effect of denoising in DN and MS COCO.

Table 8 shows the performance maintenance rate by comparing OD performance (mAP) for the original images and OD performance (mAP) for bicubic upsampling images for each degradation method. Note that the performance maintenance rate is also in the reverse order of the performance enhancement rate for the Widerface and MS COCO datasets.

The enhancement rate of OD performance generally follows a similar trend to that of the PSNR index, as shown in Table 9. The OD performance benchmark for the original MS COCO and WiderFace datasets is summarized in Table 10.

## 7. Discussions

### 7.1. Applicability of SR and OD End-to-End Models

As discussed in Section 1, this study aimed to find a method to overcome the limitations of detection models, which have a significantly low detection performance for LR input or small object targets. Several experiments have shown that the SR model mitigates the detection performance degradation problem due to LR input and dramatically enhances detection performance for small objects. In particular, the effect is maximized in models with high detection performance for large objects but low detection performance for small objects due to the structural characteristics of each model, e.g., DETR. We believe that this study has provided us with an opportunity to consider the possible effects of incorporating the SR model and detection model into an end-to-end structure. While most SR methods were confirmed to increase OD performance, several SR methods negatively affected OD performance. This is presumed to be a result of the data dependency (e.g., domain) used in the training.

### 7.2. Compatibility of GAN-Based SR Method and Detection Model

We found that the overall performance enhancement rate of EfficientDet was low, and the performance enhancement rate of ESRGAN was significantly reduced compared to that of RRDB in EfficientDet, as shown in Table 3. Comparing EfficientDet to other general OD models, two differences are observed: weighted fusion is performed by weighting each scale feature map in the feature map fusion process, and the depth-wise separable convolution proposed by MobileNet [102] is used. In addition, although ESRGAN, which is an adversarial learning-based SR model, generates detailed textures, it is limited in that it is a fake texture that looks like the real image [71].

Based on this fact, it is considered that two main factors influenced the lower performance enhancement rate of ESRGAN. First, the feature extracted from the fake texture generated by the GAN-based SR model differs from the real image feature, which can be a problem in the weighted fusion process. Second, there is an architectural distinction between classic and depth-wise separable convolution. To be specific, depth-wise separable convolution performs a convolution operation on a separate kernel for each channel’s feature map before applying point-wise convolution to combine the pixels of all channels into a single pixel. Classic convolution looks at information from all channels on the feature map from the beginning of the operation. As a result, we can assume that the effect of preventing the extraction of incorrect features by smoothing noise on the feature map has occurred. Therefore, when using a depth-wise separable convolution-based detector to solve real-world problems, the effect of improving detection performance using the GAN-based SR model may fall short of expectations.

### 7.3. Super-Resolution and Instance Segmentation

In this study, we found that the OD model’s detection performance was enhanced using SR as an input to OD models. We expect that this configuration is also applicable to instance segmentation, which is a dense OD task. Since instance segmentation is a task that models the relationship between pixels in the region corresponding to the instance, it is expected that there will be performance leverage by the GAN-based method rather than the simple interpolation-based SR method.

### 7.4. Indirect Quantitative Performance Evaluation of GAN Using OD

Since GANs generally have the effect of improving visual quality, quantitative performance evaluation is not easy. Currently, representative evaluation methods for GANs include Frechet Inception Distance that aims to measure the similarity of a feature map, and a turing test and Mean Opinion Score test that investigates the visual quality by asking a question to people. The detection performance improvement by the GAN-based SR model was quantitatively analyzed in this study using the performance enhancement rate. Currently, representative evaluation methods for GANs include Frechet inception distance (FID) that aims to measure the similarity of a feature map, and a Turing test and mean opinion score (MOS) test that investigates the visual quality by asking a question to people.

### 7.5. Analysis of the Latest Transformer-Based SR Models

Leveraging the attention mechanism of the Transformer architecture, the newest SR models exhibit superior performance (PSNR/SSIM) compared to CNN-based models in addressing the simplest form of degradation, BI, even when trained on PNG format data. However, in dealing with the most challenging degradation method, DN, these models show lower performance (PSNR/SSIM) relative to previous models. This reduction in performance is speculated to arise from differences in the format of image data.

### 7.6. Analysis of the Latest Transformer-Based OD Models

Since the advent of DETR, a plethora of new OD models based on DETR have been extensively researched, demonstrating superior performance compared to traditional models. Consequently, while deep learning-based SR methods exhibit higher performance than the baseline SR method of bicubic interpolation, the overall rate of performance improvement is relatively low. This is because, even at the baseline, these models have shown commendable performance when compared to alternative models.

## 8. Conclusions

This paper has focused on addressing the limitations of OD models, where detection performance is degraded if an input image has a low quality, with noise or low resolution. We have examined a solution to this problem by properly fusing the OD and SR models, and we investigated various SR and OD models to find the best combination. By selecting several SR and OD models, we performed experiments to improve OD performance. In addition, we employed well-known degradation methods to simulate real-world low-quality images. Extensive quantitative experiments were performed using the MS COCO dataset in a general OD experiment and the Widerface dataset in a face detection experiment. The experimental results confirm that detection performance for small objects was significantly improved in most OD models (9.4%), and a suitable combination for fusing SR and OD models was sought via quantitative analysis. Our experimental results are expected to be easily extended to various fields of real computer vision such as instance segmentation. Future research will focus on addressing the performance degradation in SR and OD observed in certain experiments by combining various modalities such as language and text.

## Figures and Tables

**Table 1:** Training details for the SR models selected as an experimental group. UM: upsampling method, UL: upsampling location, Key feature: key feature of the architecture.

SR Model	Publication	Loss Function	UM	UL	Key Feature
DRRN [49]	CVPR’17	MSE	Bicubic	Pre	Recursive
EDSR [42]	CVPRW’17	L1	Sub-pixel	Post	Scaling model
DBPN [36]	CVPR’18	L1	Deconv	Iterative	Dense and Back-projection
MSRN [66]	ECCV’18	L1	Sub-pixel	Post	Multi-path and Multi-scale receptive field
RCAN [71]	ECCV’18	L1	Sub-pixel	Post	Channel attention
RRDB [54]	ECCVW’18	L1	Sub-pixel	Post	Residual-in-residual dense block
ESRGAN [54]	ECCVW’18	L1 + GAN + $L_{perceptual}$	Sub-pixel	Post	RRDB + Adversarial learning
HAT [74]	CVPR’23	L1	Sub-pixel	Post	Residual Hybrid Attention
DAT [75]	ICCV’23	L1	Sub-pixel	Post	Dual Aggregation Block

**Table 2:** Average object detection performance enhancement rate of each SR model for the MS COCO 2017 validation set [3]. The enhancement rate was calculated by setting bicubic interpolation as the baseline, and AP-all performance was averaged for all object detection models. (**Bold**: the highest value, underline: the second-highest value, (n.): Relative Index).

The Average Performance Enhancement Rate for MS COCO [3]					
SR Model	**BI**	**BD**	**DN**	**All**	Average Relative Index
DRRN [49]	3.1 (0.23)	1.2 (0.12)	16.3 (0.03)	6.9	0.13
DBPN [36]	10.3 (0.78)	6.2 (0.64)	382.6 (0.87)	133	0.76
MSRN [66]	10.2 (0.77)	5.9 (0.61)	384.1 (0.88)	133.4	0.75
RCAN [71]	11.8 (0.90)	7 (0.72)	401 (0.91)	139.9	0.57
EDSR [42]	**13.1 (1.00)**	7.6 (0.79)	**436 (1.00)**	152.2	0.93
RRDB [54]	11 (0.83)	8.1 (0.84)	281.9 (0.64)	100.3	0.77
ESRGAN [54]	12.1 (0.92)	**9.6 (1.00)**	404.2 (0.92)	142	0.95
HAT [74]	−8.8 (-)	7.8 (0.81)	366.7 (0.84)	121.9	-
DAT [75]	−11.7 (-)	6.7 (0.69)	406 (0.93)	133.7	-
Average	5.7	6.7	342.1	118.1	-

**Table 3:** The enhancement rate of object detection performance of each SR model for the MS COCO dataset [3]. On average, AP-small is higher than AP-all. AP-all: enhancement rate for all objects, AP-small: enhancement rate for small objects.

The Enhancement Rate for MS COCO [3]																
Detector	EfficientDet		YOLOv3		Faster R-CNN		RetinaNet		DETR		DINO		Co-DETR		Average	
AP	AP-all	AP-small	AP-all	AP-small	AP-all	AP-small	AP-all	AP-small	AP-all	AP-small	AP-all	AP-small	AP-all	AP-small	AP-all	AP-small
DRRN [49]	−2.0	12.1	25.6	23.4	11.4	20.1	18.5	55.8	6.2	24.4	−5.7	−6.9	−5.7	−7.8	6.9	17.3
DBPN [36]	40.5	71.0	137.9	127.1	172.9	199.0	259.6	339.1	315.5	306.3	1.7	4	3.3	4.3	133.1	150.1
MSRN [66]	39.6	71.3	139.7	126.5	176.0	219.2	253.8	306.4	318.8	288.3	2.4	0.4	3.4	3.3	133.4	145.1
RCAN [71]	42.2	82.6	144.6	133.9	182.9	207.1	270.3	343.5	331.3	327.7	3.3	3.7	5	6.9	139.9	157.9
EDSR [42]	44.9	86.8	151.7	140.3	195.9	224.8	290.4	381.3	371.8	352.6	4.9	6.6	6.1	10.1	152.2	171.8
RRDB [54]	44.3	82.3	149.3	137.0	192.6	217.0	280.1	344.6	346.9	348.6	3	3	5.7	8.1	146	162.9
ESRGAN [54]	37.8	55.5	140.4	108.4	189.1	190.8	284.0	301.0	340.6	273.4	0.5	−7.4	1.4	−5	142	131
HAT [74]	46.1	103.9	121.8	113.3	160.2	205.8	238	224.1	281.3	291.9	2.2	3.5	3.7	7.6	121.9	135.7
DAT [75]	46.6	95.1	133.6	115.3	170.9	187.4	254.6	302.2	329.5	288.9	−0.3	−2.3	0.9	0	133.7	140.9
Average	37.8	73.4	127.2	113.9	161.3	185.7	238.8	288.7	293.5	278	1.3	0.5	2.6	3.1	123.2	134.7

**Table 4:** Average object detection performance enhancement rate of each SR model on the Widerface validation set [13]. The enhancement rate was calculated by using bicubic interpolation as the baseline and averaging AP performance across all object detection models. (**Bold**: the highest value, underline: the second-highest value).

The Enhancement Rate for Widerface [13]				
SR Model	**BI**	**BD**	**DN**	All
DRRN [49]	−0.77	−0.23	11.9	3.63
ESRGAN [54]	−1.1	−0.23	47.9	15.52
DBPN [36]	0.13	0.83	55.07	18.68
MSRN [66]	0	0.87	55.77	18.88
RRDB [54]	0.17	0.8	56.03	19
RCAN [71]	0.13	0.83	56	18.99
EDSR [42]	0.13	0.97	**57.2**	**19.43**
HAT [74]	−1.6	**1.13**	56.73	18.75
DAT [75]	**0.6**	0.93	56	19.18
Average	−0.26	0.66	50.29	16.9

**Table 5:** Results of comparing the AP for the Wider Face validation hard set of all object detection models by training RRDB [54] and ESRGAN [54] with DIV2K [99] and Widerface training set [13], respectively.

The AP for Widerface [13]				
Training data	DIV2K		Widerface	
SR Model	RRDB [54]	ESRGAN [54]	RRDB	ESRGAN
with BI	0.596	0.579	0.605	0.606
with BD	0.612	0.605	0.620	0.627
with DN	0.399	0.368	0.418	0.441
Average	0.536	0.517	0.548	0.558

**Table 6:** The AP performance maintenance rate of an object detection model for a bicubic upsampling image.

The AP Maintenance Rate of Detectors for MS COCO [3]								
Detector	EfficientDet	YOLOv3	DETR	Faster R-CNN	RetinaNet	DINO	Co-DETR	Average
Maintenance rate	60.1%	58.0%	52.5%	50.8%	49.4%	71.9%	71.9%	59.2%

**Table 7:** Performance enhancement rate of each object detection model for the easy, medium, and hard sets while each degradation is shown, including the case of using all SR models for the Widerface validation set [13].

The Enhancement Rate for Widerface [13]				
Detector	Easy	Medium	Hard	Average
EfficientDet	1.62	3.07	7.43	4.04
Faster R-CNN	5.86	7.84	11.74	8.48
RetinaNet	9.8	13.1	20.87	14.59
YOLOv3	22.27	34.06	56.41	37.58
DETR	27.06	30.03	36.64	31.24
DINO	7.3	8.52	11.12	8.98
Co-DETR	10.63	12.42	17.13	13.39
Detector	**BI**	**BD**	**DN**	Average
EfficientDet	0.74	2.84	8.54	4.04
Faster R-CNN	−0.57	1.06	24.96	8.48
RetinaNet	0.34	0.92	42.5	14.59
YOLOv3	0.33	2.38	110.03	37.58
DETR	−2.08	−4.11	99.92	31.24
DINO	−0.29	0.64	26.58	8.98
Co-DETR	−0.2	0.89	39.49	13.39

**Table 8:** The baseline performance maintenance rate for each degradation of the detector model for the Wider Face validation set [13].

Detector	BI	BD	DN	All
YOLOv3	96.7	95.8	50.5	81
RetinaNet	96.4	96.7	59.5	84.2
Faster R-CNN	96.8	96.3	66.7	86.6
EfficientDet	96.7	95.4	80.3	90.8
DINO	97.3	97.2	65.8	86.8
Co-DETR	96.7	96.5	61	84.7
Average	98	98.1	62.1	86.1

**Table 9:** Set5 [101] benchmark results of the experimental SR models demonstrated using the PSNR/SSIM [12] method.

SR Model	Scale	BI	BD	DN
Bicubic (baseline)	2	34.42/0.9395	29.73/0.8583	24.64/0.5283
ESRGAN [54]	2	35.27/0.9310	34.46/0.9181	27.98/0.8427
DRRN [49]	2	35.54/0.9444	34.89/0.9306	29.57/0.7348
DBPN [36]	2	36.87/0.9545	35.82/0.9393	30.67/0.8615
MSRN [66]	2	36.93/0.9549	35.82/0.9391	30.77/0.8634
RCAN [71]	2	37.05/0.9553	35.99/0.9402	30.87/0.8660
EDSR [42]	2	37.13/0.9557	36.00/0.9404	30.89/0.8665
RRDB [54]	2	37.18/0.9558	36.12/0.9411	30.93/0.8677
DAT [75]	2	37.76/0.9597	36.05/0.9411	28.89/0.7649
HAT [74]	2	37.98/0.9604	36.18/0.9423	29.50/0.7963
Bicubic (baseline)	4	27.68/0.8102	28.25/0.8077	22.93/0.5380
ESRGAN	4	27.71/0.8129	29.83/0.8434	24.96/0.7276
DRRN	4	28.53/0.8369	30.35/0.8609	25.10/0.6917
DBPN	4	29.47/0.8682	31.70/0.8893	26.63/0.7790
MSRN	4	29.53/0.8674	31.60/0.8882	26.67/0.7795
RCAN	4	29.74/0.8715	31.96/0.8927	26.81/0.7850
EDSR	4	29.84/0.8744	31.96/0.8928	26.87/0.7861
RRDB	4	29.90/0.8752	32.03/0.8944	26.88/0.7869
DAT	4	31.51/0.8844	31.39/0.8841	25.17/0.6863
HAT	4	31.63/0.8859	31.48/0.8850	26.08/0.7736

**Table 10:** OD performance (mAP) benchmark of representative DL-based OD models. Note that OD model was used with pretrained weights.

MS COCO [3] Benchmark (mAP)				
Detector	Small	Medium	Large	All
Faster R-CNN [6]	0.252	0.456	0.546	0.420
RetinaNet [84]	0.240	0.443	0.522	0.404
YOLOv3 [88]	0.270	0.492	0.576	0.438
DETR [94]	0.207	0.459	0.611	0.420
EfficientDet [4]	0.400	0.581	0.679	0.544
DINO [96]	0.414	0.619	0.736	0.580
Co-DETR [97]	0.425	0.627	0.751	0.589
Wider Face [13] Benchmark (mAP)				
Detector	Easy	Medium	Hard	Average
Faster R-CNN	0.928	0.897	0.691	0.839
RetinaNet	0.941	0.910	0.691	0.847
YOLOv3	0.941	0.928	0.823	0.897
DETR	0.776	0.781	0.595	0.717
EfficientDet	0.943	0.926	0.613	0.827
DINO	0.923	0.898	0.733	0.851
Co-DETR	0.942	0.924	0.757	0.874

## Data Availability

Data are contained within the article.

## References

[1] Deng J., Dong W., Socher R., Li L.J., Li K., Fei-Fei L. ImageNet: A Large-Scale Hierarchical Image Database. Proceedings of the IEEE Conference on Computer Vision and Pattern Recognition.

[2] Everingham M., Van Gool L., Williams C.K., Winn J., Zisserman A. (2010). The pascal visual object classes (voc) challenge. Int. J. Comput. Vis..

[3] Lin T.Y., Maire M., Belongie S., Hays J., Perona P., Ramanan D., Dollár P., Zitnick C.L. (2014). Microsoft COCO: Common objects in context. Computer Vision—ECCV 2014, Proceedings of the 13th European Conference, Zurich, Switzerland, 6–12 September 2014.

[4] Tan M., Pang R., Le Q.V. Efficientdet: Scalable and efficient object detection. Proceedings of the IEEE Conference on Computer Vision and Pattern Recognition.

[5] Anwar A., Raychowdhury A. (2020). Masked Face Recognition for Secure Authentication. arXiv.

[6] Ren S., He K., Girshick R., Sun J. (2015). Faster r-cnn: Towards real-time object detection with region proposal networks. Advances in Neural Information Processing Systems.

[7] Redmon J., Divvala S., Girshick R., Farhadi A. You only look once: Unified, real-time object detection. Proceedings of the IEEE Conference on Computer Vision and Pattern Recognition.

[8] Liu L., Ouyang W., Wang X., Fieguth P., Chen J., Liu X., Pietikäinen M. (2020). Deep learning for generic object detection: A survey. Int. J. Comput. Vis..

[9] Jiao L., Zhang F., Liu F., Yang S., Li L., Feng Z., Qu R. (2019). A survey of deep learning-based object detection. IEEE Access.

[10] Bai Y., Zhang Y., Ding M., Ghanem B. Sod-mtgan: Small object detection via multi-task generative adversarial network. Proceedings of the 15th European Conference.

[11] Haris M., Shakhnarovich G., Ukita N., Mantoro T., Lee M., Ayu M.A., Wong K.W., Hidayanto A.N. (2021). Task-Driven Super Resolution: Object Detection in Low-Resolution Images. Neural Information Processing, Proceedings of the 28th International Conference, ICONIP 2021.

[12] Wang Z., Bovik A.C., Sheikh H.R., Simoncelli E.P. (2004). Image quality assessment: From error visibility to structural similarity. IEEE Trans. Image Process..

[13] Yang S., Luo P., Loy C.C., Tang X. WIDER FACE: A Face Detection Benchmark. Proceedings of the IEEE Conference on Computer Vision and Pattern Recognition.

[14] Pang Y., Cao J., Wang J., Han J. (2019). JCS-Net: Joint Classification and Super-Resolution Network for Small-Scale Pedestrian Detection in Surveillance Images. IEEE Trans. Inf. Forensics Secur..

[15] Wang L., Li D., Zhu Y., Tian L., Shan Y. Dual super-resolution learning for semantic segmentation. Proceedings of the IEEE/CVF Conference on Computer Vision and Pattern Recognition.

[16] Xiao J., Jiang X., Zheng N., Yang H., Yang Y., Yang Y., Li D., Lam K.M. (2023). Online video super-resolution with convolutional kernel bypass grafts. IEEE Trans. Multimed..

[17] Ju Y., Jian M., Wang C., Zhang C., Dong J., Lam K.M. (2023). Estimating high-resolution surface normals via low-resolution photometric stereo images. IEEE Trans. Circuits Syst. Video Technol..

[18] Wang B., Lu T., Zhang Y. Feature-Driven Super-Resolution for Object Detection. Proceedings of the 2020 5th International Conference on Control, Robotics and Cybernetics (CRC).

[19] Zheng S., Wu Y., Jiang S., Lu C., Gupta G. Deblur-YOLO: Real-Time Object Detection with Efficient Blind Motion Deblurring. Proceedings of the 2021 International Joint Conference on Neural Networks (IJCNN).

[20] Noh J., Bae W., Lee W., Seo J., Kim G. Better to follow, follow to be better: Towards precise supervision of feature super-resolution for small object detection. Proceedings of the IEEE/CVF International Conference on Computer Vision.

[21] Yang J., Wright J., Huang T.S., Ma Y. (2010). Image super-resolution via sparse representation. IEEE Trans. Image Process..

[22] Timofte R., De Smet V., Van Gool L. (2014). A+: Adjusted anchored neighborhood regression for fast super-resolution. Computer Vision—ACCV 2014, Proceedings of the 12th Asian Conference on Computer Vision, Singapore, 1–5 November 2014.

[23] Schulter S., Leistner C., Bischof H. Fast and accurate image upscaling with super-resolution forests. Proceedings of the IEEE Conference on Computer Vision and Pattern Recognition.

[24] Zhang Y., Tian Y., Kong Y., Zhong B., Fu Y. Residual dense network for image super-resolution. Proceedings of the IEEE Conference on Computer Vision and Pattern Recognition.

[25] Wang X., Xie L., Dong C., Shan Y. Real-ESRGAN: Training Real-World Blind Super-Resolution with Pure Synthetic Data. Proceedings of the International Conference on Computer Vision Workshops (ICCVW).

[26] Zhang K., Liang J., Van Gool L., Timofte R. Designing a practical degradation model for deep blind image super-resolution. Proceedings of the IEEE/CVF International Conference on Computer Vision.

[27] Dong C., Loy C.C., He K., Tang X. (2015). Image super-resolution using deep convolutional networks. IEEE Trans. Pattern Anal. Mach. Intell..

[28] Kim J., Kwon Lee J., Mu Lee K. Accurate image super-resolution using very deep convolutional networks. Proceedings of the IEEE Conference on Computer Vision and Pattern Recognition.

[29] Simonyan K., Zisserman A. (2014). Very deep convolutional networks for large-scale image recognition. arXiv.

[30] Goodfellow I., Pouget-Abadie J., Mirza M., Xu B., Warde-Farley D., Ozair S., Courville A., Bengio Y. Generative adversarial nets. Proceedings of the 27th International Conference on Neural Information Processing Systems.

[31] Wang Z., Liu D., Yang J., Han W., Huang T. Deep Networks for Image Super-Resolution with Sparse Prior. Proceedings of the IEEE International Conference on Computer Vision.

[32] Zheng H., Ji M., Wang H., Liu Y., Fang L. CrossNet: An End-to-end Reference-based Super Resolution Network using Cross-scale Warping. Proceedings of the 15th European Conference.

[33] Dong C., Loy C.C., Tang X. Accelerating the super-resolution convolutional neural network. Proceedings of the 14th European Conference.

[34] Shi W., Caballero J., Huszár F., Totz J., Aitken A.P., Bishop R., Rueckert D., Wang Z. Real-time single image and video super-resolution using an efficient sub-pixel convolutional neural network. Proceedings of the IEEE Conference on Computer Vision and Pattern Recognition.

[35] Lai W.S., Huang J.B., Ahuja N., Yang M.H. Deep laplacian pyramid networks for fast and accurate super-resolution. Proceedings of the IEEE Conference on Computer Vision and Pattern Recognition (CVPR).

[36] Haris M., Shakhnarovich G., Ukita N. Deep back-projection networks for super-resolution. Proceedings of the IEEE Conference on Computer Vision and Pattern Recognition (CVPR).

[37] He K., Zhang X., Ren S., Sun J. Delving deep into rectifiers: Surpassing human-level performance on imagenet classification. Proceedings of the IEEE International Conference on Computer Vision.

[38] Zhang K., Zuo W., Zhang L. Learning a single convolutional super-resolution network for multiple degradations. Proceedings of the IEEE Conference on Computer Vision and Pattern Recognition (CVPR).

[39] Kingma D.P., Ba J. (2014). Adam: A method for stochastic optimization. arXiv.

[40] He K., Zhang X., Ren S., Sun J. Deep residual learning for image recognition. Proceedings of the IEEE Conference on Computer Vision and Pattern Recognition.

[41] Ledig C., Theis L., Huszár F., Caballero J., Cunningham A., Acosta A., Aitken A., Tejani A., Totz J., Wang Z. Photo-realistic single image super-resolution using a generative adversarial network. Proceedings of the IEEE Conference on Computer Vision and Pattern Recognition (CVPR).

[42] Lim B., Son S., Kim H., Nah S., Mu Lee K. Enhanced deep residual networks for single image super-resolution. Proceedings of the IEEE Conference on Computer Vision and Pattern Recognition Workshops.

[43] Timofte R., Agustsson E., Van Gool L., Yang M.H., Zhang L. Ntire 2017 challenge on single image super-resolution: Methods and results. Proceedings of the IEEE Conference on Computer Vision and Pattern Recognition Workshops.

[44] Han W., Chang S., Liu D., Yu M., Witbrock M., Huang T.S. Image super-resolution via dual-state recurrent networks. Proceedings of the IEEE Conference on Computer Vision and Pattern Recognition (CVPR).

[45] Wang Z., Chen J., Hoi S.C. (2020). Deep learning for image super-resolution: A survey. IEEE Trans. Pattern Anal. Mach. Intell..

[46] Kim J., Kwon Lee J., Mu Lee K. Deeply-recursive convolutional network for image super-resolution. Proceedings of the IEEE Conference on Computer Vision and Pattern Recognition.

[47] Tai Y., Yang J., Liu X., Xu C. Memnet: A persistent memory network for image restoration. Proceedings of the IEEE International Conference on Computer Vision.

[48] Li Z., Yang J., Liu Z., Yang X., Jeon G., Wu W. Feedback network for image super-resolution. Proceedings of the IEEE Conference on Computer Vision and Pattern Recognition.

[49] Tai Y., Yang J., Liu X. Image super-resolution via deep recursive residual network. Proceedings of the IEEE Conference on Computer Vision and Pattern Recognition (CVPR).

[50] Liu D., Wen B., Fan Y., Loy C.C., Huang T.S. Non-local recurrent network for image restoration. Proceedings of the 32nd Conference on Neural Information Processing Systems (NIPS 2018).

[51] Huang G., Liu Z., Van Der Maaten L., Weinberger K.Q. Densely connected convolutional networks. Proceedings of the IEEE Conference on Computer Vision and Pattern Recognition (CVPR).

[52] Tong T., Li G., Liu X., Gao Q. Image super-resolution using dense skip connections. Proceedings of the IEEE International Conference on Computer Vision.

[53] Timofte R., Gu S., Wu J., Van Gool L. Ntire 2018 challenge on single image super-resolution: Methods and results. Proceedings of the IEEE Conference on Computer Vision and Pattern Recognition Workshops.

[54] Wang X., Yu K., Wu S., Gu J., Liu Y., Dong C., Qiao Y., Change Loy C. Esrgan: Enhanced super-resolution generative adversarial networks. Proceedings of the European Conference on Computer Vision.

[55] Ahn N., Kang B., Sohn K.A. Fast, accurate, and lightweight super-resolution with cascading residual network. Proceedings of the European Conference on Computer Vision.

[56] Sajjadi M.S., Scholkopf B., Hirsch M. Enhancenet: Single image super-resolution through automated texture synthesis. Proceedings of the IEEE International Conference on Computer Vision.

[57] Park S.J., Son H., Cho S., Hong K.S., Lee S. Srfeat: Single image super-resolution with feature discrimination. Proceedings of the European Conference on Computer Vision.

[58] Zhang Z., Wang Z., Lin Z., Qi H. Image super-resolution by neural texture transfer. Proceedings of the IEEE Conference on Computer Vision and Pattern Recognition.

[59] Gulrajani I., Ahmed F., Arjovsky M., Dumoulin V., Courville A.C. Improved training of wasserstein gans. Proceedings of the Advances in Neural Information Processing Systems.

[60] Dosovitskiy A., Fischer P., Ilg E., Hausser P., Hazirbas C., Golkov V., Van Der Smagt P., Cremers D., Brox T. Flownet: Learning optical flow with convolutional networks. Proceedings of the IEEE International Conference on Computer Vision.

[61] Ronneberger O., Fischer P., Brox T. (2015). U-net: Convolutional networks for biomedical image segmentation. Medical Image Computing and Computer-Assisted Intervention—MICCAI 2015, Proceedings of the 18th International Conference, Munich, Germany, 5–9 October 2015.

[62] Bruhn A., Weickert J., Schnörr C. (2005). Lucas/Kanade meets Horn/Schunck: Combining local and global optic flow methods. Int. J. Comput. Vis..

[63] Yang F., Yang H., Fu J., Lu H., Guo B. Learning texture transformer network for image super-resolution. Proceedings of the IEEE/CVF Conference on Computer Vision and Pattern Recognition.

[64] Vaswani A., Shazeer N., Parmar N., Uszkoreit J., Jones L., Gomez A.N., Kaiser Ł., Polosukhin I. Attention is all you need. Proceedings of the 31st International Conference on Neural Information Processing Systems.

[65] Gregor K., LeCun Y. Learning fast approximations of sparse coding. Proceedings of the 27th International Conference on International Conference on Machine Learning.

[66] Li J., Fang F., Mei K., Zhang G. Multi-scale residual network for image super-resolution. Proceedings of the European Conference on Computer Vision.

[67] Hu Y., Gao X., Li J., Huang Y., Wang H. (2018). Single image super-resolution via cascaded multi-scale cross network. arXiv.

[68] Ren H., El-Khamy M., Lee J. Image super resolution based on fusing multiple convolution neural networks. Proceedings of the IEEE Conference on Computer Vision and Pattern Recognition Workshops.

[69] Hui Z., Wang X., Gao X. Fast and accurate single image super-resolution via information distillation network. Proceedings of the IEEE Conference on Computer Vision and Pattern Recognition (CVPR).

[70] Hu J., Shen L., Sun G. Squeeze-and-excitation networks. Proceedings of the IEEE Conference on Computer Vision and Pattern Recognition (CVPR).

[71] Zhang Y., Li K., Li K., Wang L., Zhong B., Fu Y. Image super-resolution using very deep residual channel attention networks. Proceedings of the European Conference on Computer Vision.

[72] Dai T., Cai J., Zhang Y., Xia S.T., Zhang L. Second-order attention network for single image super-resolution. Proceedings of the IEEE Conference on Computer Vision and Pattern Recognition.

[73] Zhang W., Zhao W., Li J., Zhuang P., Sun H., Xu Y., Li C. (2024). CVANet: Cascaded visual attention network for single image super-resolution. Neural Netw..

[74] Chen X., Wang X., Zhou J., Qiao Y., Dong C. Activating more pixels in image super-resolution transformer. Proceedings of the IEEE/CVF Conference on Computer Vision and Pattern Recognition.

[75] Chen Z., Zhang Y., Gu J., Kong L., Yang X., Yu F. Dual aggregation transformer for image super-resolution. Proceedings of the IEEE/CVF International Conference on Computer Vision.

[76] Shocher A., Cohen N., Irani M. “Zero-shot” super-resolution using deep internal learning. Proceedings of the IEEE Conference on Computer Vision and Pattern Recognition (CVPR).

[77] Maeda S. Unpaired Image Super-Resolution using Pseudo-Supervision. Proceedings of the IEEE Conference on Computer Vision and Pattern Recognition.

[78] Girshick R., Donahue J., Darrell T., Malik J. Rich feature hierarchies for accurate object detection and semantic segmentation. Proceedings of the IEEE Conference on Computer Vision and Pattern Recognition.

[79] He K., Gkioxari G., Dollár P., Girshick R. Mask r-cnn. Proceedings of the IEEE International Conference on Computer Vision.

[80] Uijlings J.R., Van De Sande K.E., Gevers T., Smeulders A.W. (2013). Selective search for object recognition. Int. J. Comput. Vis..

[81] He K., Zhang X., Ren S., Sun J. (2015). Spatial pyramid pooling in deep convolutional networks for visual recognition. IEEE Trans. Pattern Anal. Mach. Intell..

[82] Dai J., Li Y., He K., Sun J. (2016). R-fcn: Object detection via region-based fully convolutional networks. Adv. Neural Inf. Process. Syst..

[83] Lin T.Y., Dollár P., Girshick R., He K., Hariharan B., Belongie S. Feature pyramid networks for object detection. Proceedings of the IEEE Conference on Computer Vision and Pattern Recognition (CVPR).

[84] Lin T.Y., Goyal P., Girshick R., He K., Dollár P. Focal loss for dense object detection. Proceedings of the IEEE International Conference on Computer Vision.

[85] Rezatofighi H., Tsoi N., Gwak J., Sadeghian A., Reid I., Savarese S. Generalized intersection over union: A metric and a loss for bounding box regression. Proceedings of the IEEE Conference on Computer Vision and Pattern Recognition.

[86] Zhang S., Wen L., Bian X., Lei Z., Li S.Z. Single-shot refinement neural network for object detection. Proceedings of the IEEE Conference on Computer Vision and Pattern Recognition (CVPR).

[87] Redmon J., Farhadi A. YOLO9000: Better, faster, stronger. Proceedings of the IEEE Conference on Computer Vision and Pattern Recognition.

[88] Redmon J., Farhadi A. (2018). Yolov3: An incremental improvement. arXiv.

[89] Wang C.Y., Liao H.Y.M., Wu Y.H., Chen P.Y., Hsieh J.W., Yeh I.H. CSPNet: A new backbone that can enhance learning capability of CNN. Proceedings of the IEEE Conference on Computer Vision and Pattern Recognition Workshops.

[90] Bochkovskiy A., Wang C.Y., Liao H.Y.M. (2020). Yolov4: Optimal speed and accuracy of object detection. arXiv.

[91] Liu W., Anguelov D., Erhan D., Szegedy C., Reed S., Fu C.Y., Berg A.C. (2016). Ssd: Single shot multibox detector. Computer Vision—ECCV 2016, Proceedings of the 14th European Conference, Amsterdam, The Netherlands, 11–14 October 2016.

[92] Dosovitskiy A., Beyer L., Kolesnikov A., Weissenborn D., Zhai X., Unterthiner T., Dehghani M., Minderer M., Heigold G., Gelly S. (2020). An image is worth 16x16 words: Transformers for image recognition at scale. arXiv.

[93] Touvron H., Cord M., Douze M., Massa F., Sablayrolles A., Jégou H. Training data-efficient image transformers & distillation through attention. Proceedings of the International Conference on Machine Learning.

[94] Carion N., Massa F., Synnaeve G., Usunier N., Kirillov A., Zagoruyko S. (2020). End-to-End Object Detection with Transformers. arXiv.

[95] Zhu X., Su W., Lu L., Li B., Wang X., Dai J. (2020). Deformable detr: Deformable transformers for end-to-end object detection. arXiv.

[96] Zhang H., Li F., Liu S., Zhang L., Su H., Zhu J., Ni L.M., Shum H.Y. (2022). Dino: Detr with improved denoising anchor boxes for end-to-end object detection. arXiv.

[97] Zong Z., Song G., Liu Y. Detrs with collaborative hybrid assignments training. Proceedings of the IEEE/CVF International Conference on Computer Vision.

[98] Liu S., Li F., Zhang H., Yang X., Qi X., Su H., Zhu J., Zhang L. (2022). Dab-detr: Dynamic anchor boxes are better queries for detr. arXiv.

[99] Agustsson E., Timofte R. Ntire 2017 challenge on single image super-resolution: Dataset and study. Proceedings of the IEEE Conference on Computer Vision and Pattern Recognition Workshops.

[100] Kim K.I., Kwon Y. (2010). Single-image super-resolution using sparse regression and natural image prior. IEEE Trans. Pattern Anal. Mach. Intell..

[101] Bevilacqua M., Roumy A., Guillemot C., Alberi-Morel M.L. Low-complexity single-image super-resolution based on nonnegative neighbor embedding. Proceedings of the British Machine Vision Conference.

[102] Howard A.G., Zhu M., Chen B., Kalenichenko D., Wang W., Weyand T., Andreetto M., Adam H. (2017). Mobilenets: Efficient convolutional neural networks for mobile vision applications. arXiv.

